# Supplementary material for: CircRREB1 mediates lipid metabolism related senescent phenotypes in chondrocytes through FASN post-translational modifications
Source: Nat Commun. 2023 Aug 28;14:5242. doi: 10.1038/s41467-023-40975-7 (PMC10462713; doi:10.1038/s41467-023-40975-7)
Supplement: Supplementary file 1 — Supplementary Information [file 41467_2023_40975_MOESM1_ESM.pdf]

Supplementary Information for

CircRREB1 mediates lipid metabolism related senescent phenotypes in chondrocytes through FASN post-translational modifications

Authors and Affiliations:

Zhe Gong[#,1], Jinjin Zhu[#,1], Junxin Chen[#,1], Fan Feng[#,2], Haitao Zhang[1], Zheyuan Zhang[1], Chenxin Song[1], Kaiyu Liang[1], Shuhui Yang[1], Shunwu Fan[*,1], Xiangqian Fang[*,1], Shuying Shen[*,1]

1.Department of Orthopaedic Surgery, Sir Run Run Shaw Hospital, Medical College of Zhejiang University & Key Laboratory of Musculoskeletal System Degeneration and Regeneration Translational Research of Zhejiang Province

Sir Run Run Shaw Institute of Clinical Medicine of Zhejiang University 3 East Qingchun Road, Hangzhou, Zhejiang Province, China, 310016

2. Obstetrics and Gynecology Hospital, Kunpeng Road, Hangzhou, Zhejiang Province, China, 310016

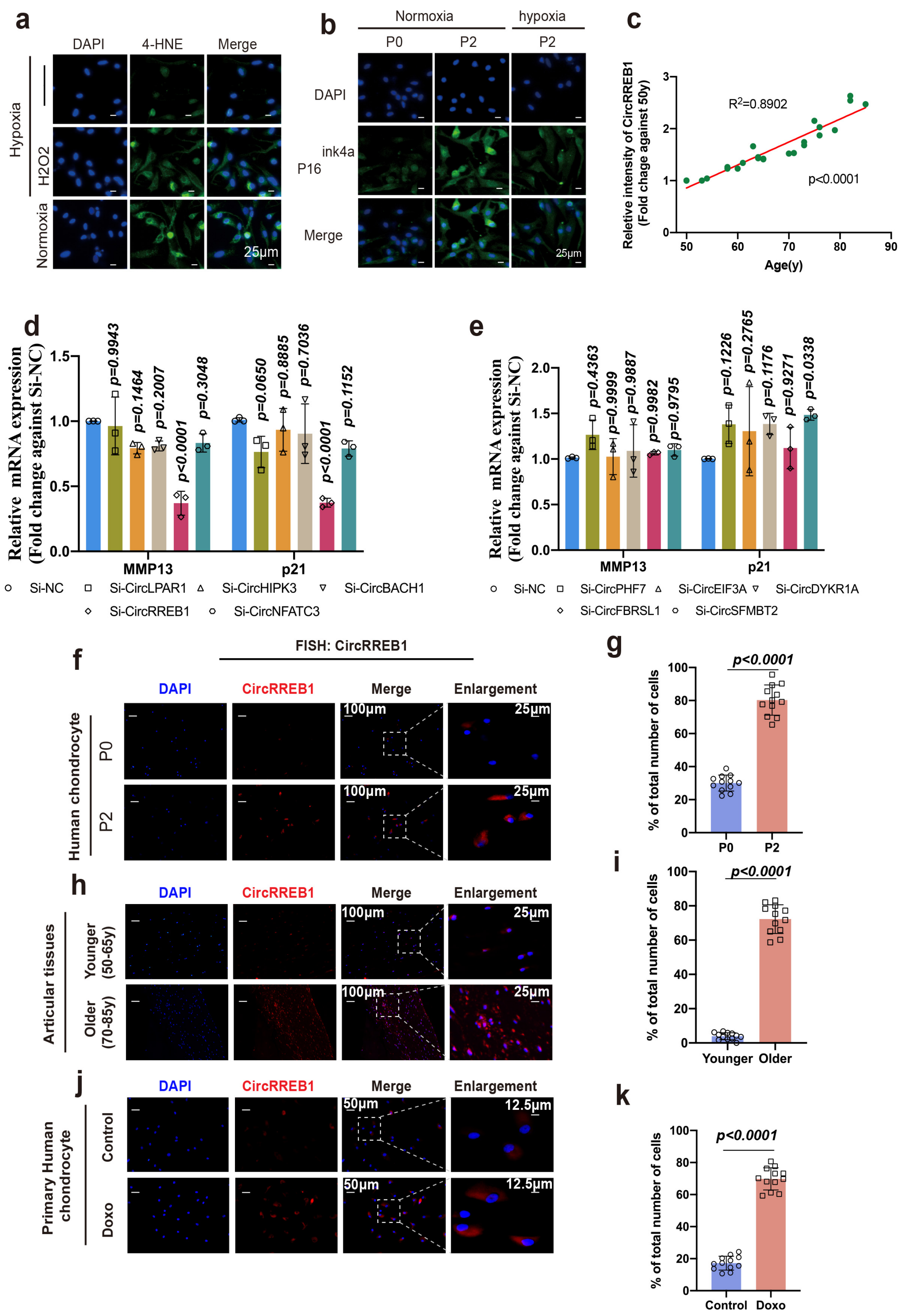

Supplementary Figure 1. a, 4-HNE expression in HCs under condition of Hypoxia and Normoxia. b, P16 expression in P0 generation chondrocyte and P2 generation chondrocyte under Hypoxia and Normoxia. c, A linear regression analysis between relative CircRREB1 intensity and age (n=24, intensity of CircRREB1 in 50y sample was used as control). d, e, *MMP13* and *p21* expression after top5 upregulated CircRNAs

knockdown and top5 downregulated CircRNAs knockdown (n=3, biological independent samples). f, RNA FISH in P0 generation chondrocyte and P2 generation chondrocyte (CircRREB1 is labeled by Cy3). g, Quantification of CircRREB1 positive cells in P0 generation chondrocyte and P2 generation (n=12, biological independent samples). h, RNA FISH in younger cartilage and older cartilage. i, Quantification of CircRREB1 in younger cartilage and older cartilage (n=12, biological independent samples). j, CircRREB1 expression in HCs treated with or without Doxo stimulation. k, Quantification of CircRREB1 in HCs treated with or without Doxo stimulation (n=12, biological independent samples). Two-sided Student's *t* test used for g, i, and k. One-way analysis of variance (ANOVA) followed by Tukey's HSD test is used for d and e. Quantitative data shown as mean ± s.d. Exact p values are shown in figures. Sacr bar for a, b: 25 μm. Scar bar for f, h: 100 μm, amplification: 25 μm. Scar bar for j: 50 μm, amplification: 12.5 μm. Source data are provided as a Source Data file.

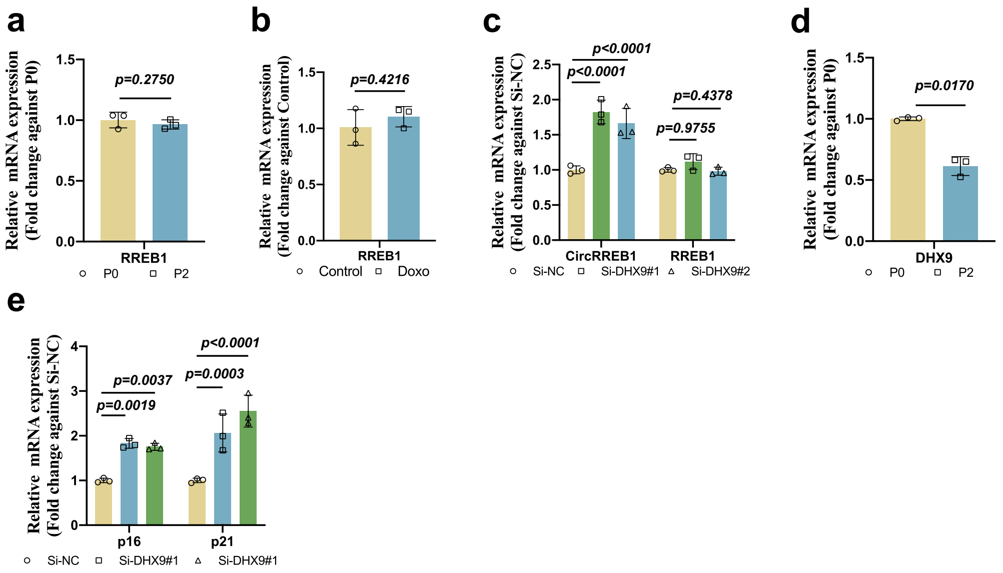

Supplementary Figure 2. CircRREB1 is regulated by DHX9. a, *RREB1* mRNA expression in P0 generation chondrocyte and P2 generation chondrocyte (n=3, biological independent samples). b, *RREB1* expression in Doxo stimulated model (n=3, biological independent samples). c, *CircRREB1* and *RREB1* expression after DHX9 knockdown in chondrocyte (n=3, biological independent samples). d, *DHX9* expression in P0 generation chondrocyte and P2 generation chondrocyte (n=3, biological

independent samples). e, Senescence associated *p16* and *p21* expression after DHX9 knockdown (n=3, biological independent samples). Two-sided Student's *t* test used for a, b, d, e. One-way analysis of variance (ANOVA) followed by Tukey's HSD test is used for c, e. Quantitative data shown as mean ± s.d. Exact p values are shown in figures. Source data are provided as a Source Data file.

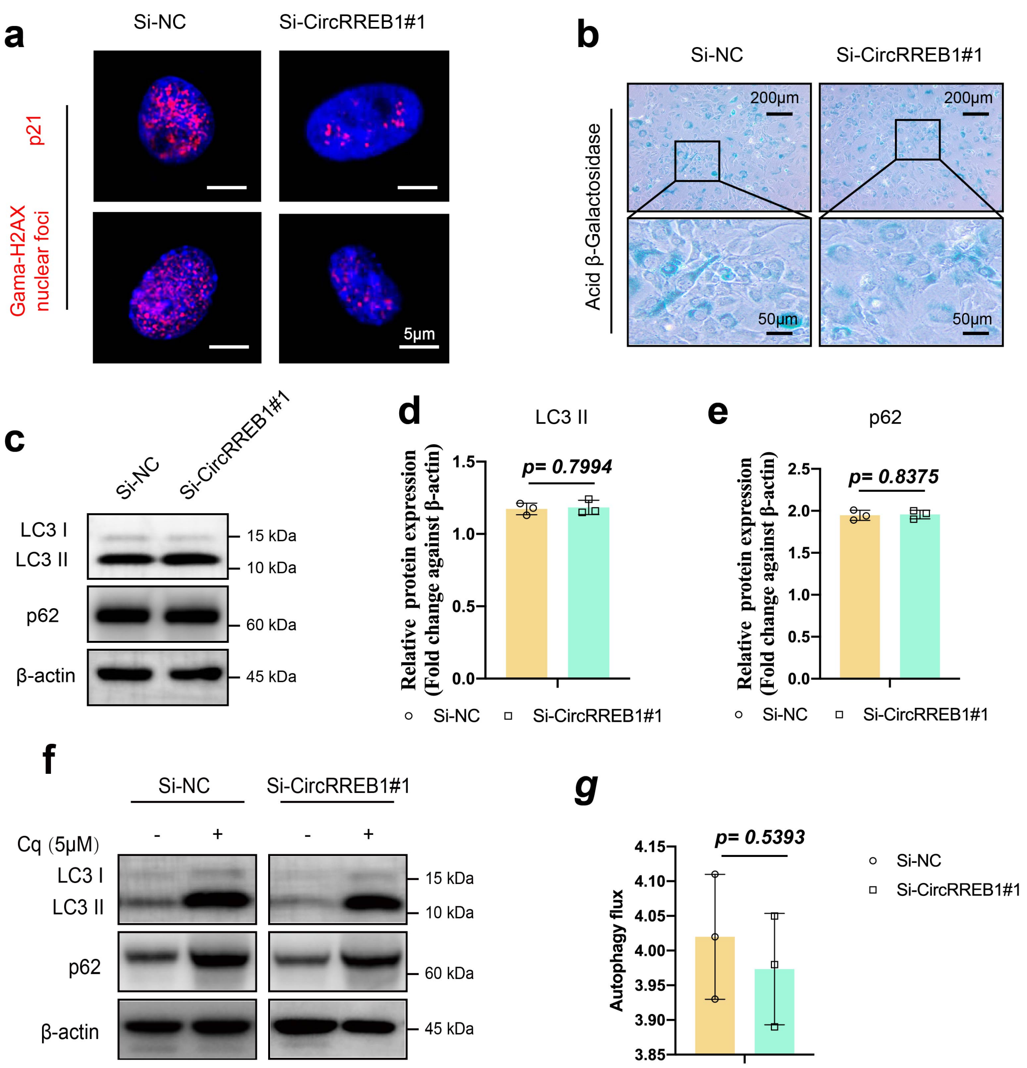

Supplementary Figure 3. a, Immunofluorescence images of p21 and gama-H2AX nuclear foci expression after CircRREB1 knockdown. Images are representative of three independent experiments. b, Acid β-Galactosidase expression after CircRREB1 knockdown. Images are representative of three independent experiments. c, LC3B and

p62 proteins expression after CircRREB1 knockdown. d, Quantification of LC3 II (n=3, biological independent samples). e, Quantification of p62 (n=3, biological independent samples). f, Chondrocytes transfected with NC or CircRREB1 SiRNA treated with or with CQ. LC3B and p62 expression are examined. g, Quantification of autophagy flux. Autophagy flux= (LC3B-II＋CQ/β-actin)/(LC3B-II-CQ/β-actin). n=3, biological independent samples. Two-sided Student's *t* test used for statistical analysis (d, e, and g). Quantitative data shown as mean ± s.d. Exact p values are shown in figures. Scar bar for a: 5 µm. Scar bar for b: 200 µm, amplification: 50 µm. Source data are provided as a Source Data file.

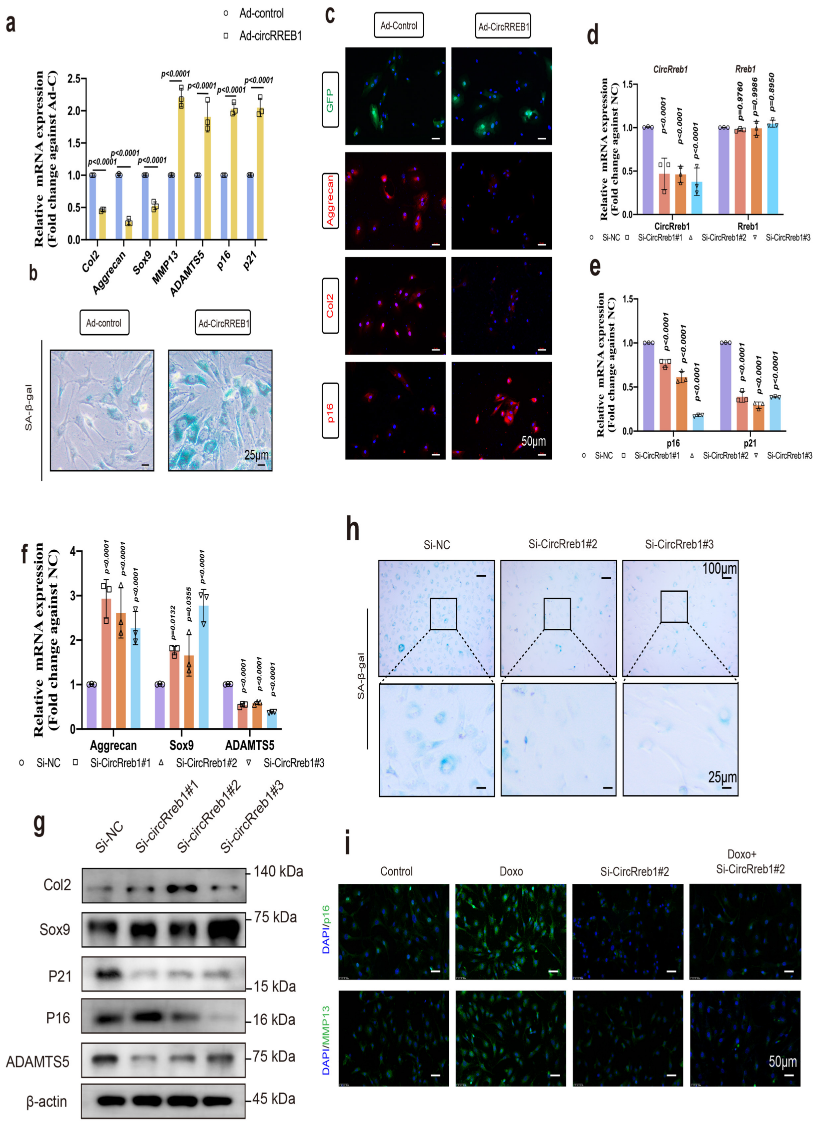

Supplementary Figure 4. a, Col2, Aggrecan, Sox9, MMP13, ADAMTS5, p16, and p21 mRNA expression in HCs infected with Ad-Control and Ad-CircRREB1 (n=3, biological independent samples). b, SA-β-Gal staining in HCs infected with Ad-Control and Ad-CircRREB1. c, Representative immunofluorescence images of GFP, Aggrecan, Col2, and p16 expression in HCs infected with Ad-Control and Ad-CircRREB1. d, CircRreb1 and Rreb1 mRNA expression in MCs infected with three CircRreb1 SiRNAs indicated by RT-qPCR (n=3, biological independent samples). e, f, *p16, p21, Aggrecan, Sox9*, and *ADAMTS5* expression in MCs infected with three CircRreb1 SiRNAs indicated by RT-qPCR (n=3, biological independent samples). g, Col2, Sox9, p21, p16, and ADAMTS5 proteins expression in MCs after CircRreb1 knockdown. Blots are representative of three independent experiments. h, SA-β-Gal staining after CircRreb1 knockdown. Images are representative of three independent experiments. i, Representative immunofluorescence images of p16 and Mmp13 in MCs infected with CircRreb1 SiRNA. Images are representative of three independent experiments. Two-sided Student's *t* test used for a. One- way analysis of variance (ANOVA) followed by Tukey's HSD test is used for d, e, and f. Quantitative data shown as mean ± s.d. Exact p values are shown in figures. Scar bar for b: 25 μm. Sar bar for c, i: 50 μm. Scar bar for h: 100 μm, amplification: 25 μm. Source data are provided as a Source Data file.

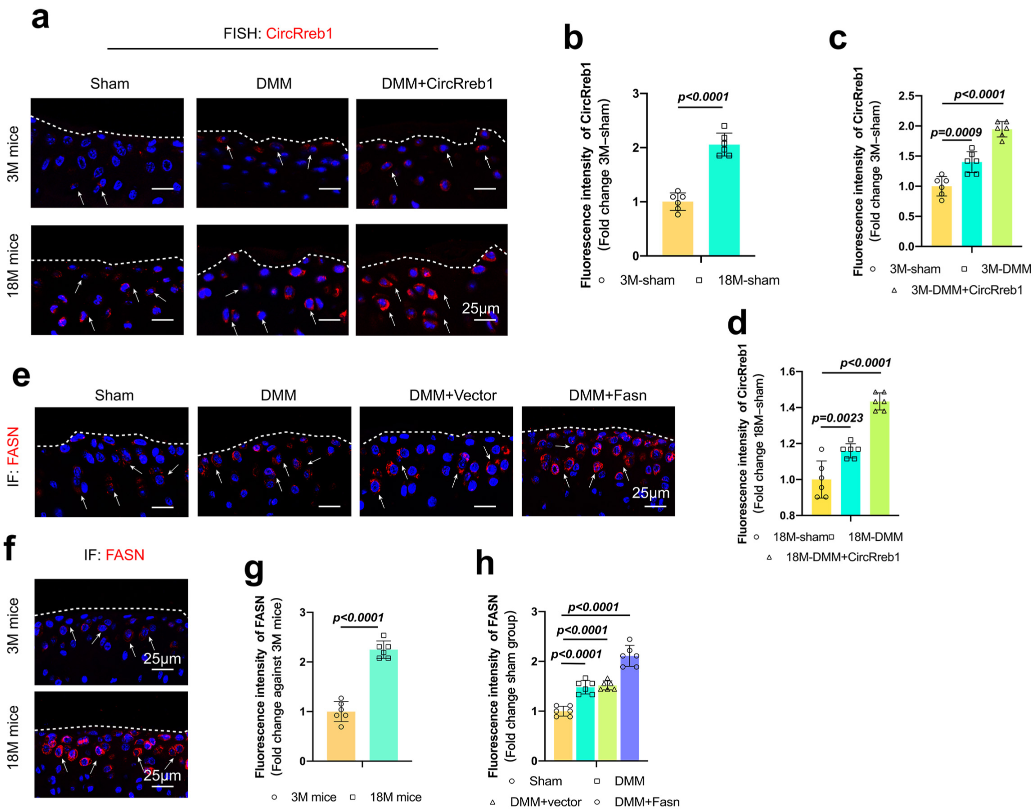

Supplementary Figure 5. a, CircRreb1 FISH staining in sham, DMM, DMM+CircRreb1 group between 3-month-old mice and 18-month-old mice (n=6, biological independent samples). b, Fluorescence intensity of CircRreb1 in 3-month-old mice and 18-month-old mice (n=6, biological independent samples). c, Fluorescence intensity of CircRreb1 in sham, DMM, DMM+CircRreb1 group in 3-month-old mice (n=6, biological independent samples). d, Fluorescence intensity of CircRreb1 in sham, DMM, DMM+CircRreb1 group in 18-month-old mice (n=6, biological independent samples). e, Representative immunofluorescence images of FASN in sham, DMM, DMM+vector, and DMM+Fasn group. f, Representative immunofluorescence images of FASN in 3-month-old mice and 18-month-old mice. g, Fluorescence intensity of FASN in 3-month-old mice and 18-month-old mice (n=6, biological independent samples). h, Fluorescence intensity of FASN in sham, DMM, DMM+vector, and DMM+Fasn group (n=6, biological independent samples). Two-sided Student's *t* test is used for b and g. One-way analysis of variance (ANOVA) followed by Tukey's HSD test is used for c, d, and h. Quantitative data shown as mean ± s.d. Exact p values are shown in figures. All scar bar: 25 μm. Source data are provided as a Source Data file.

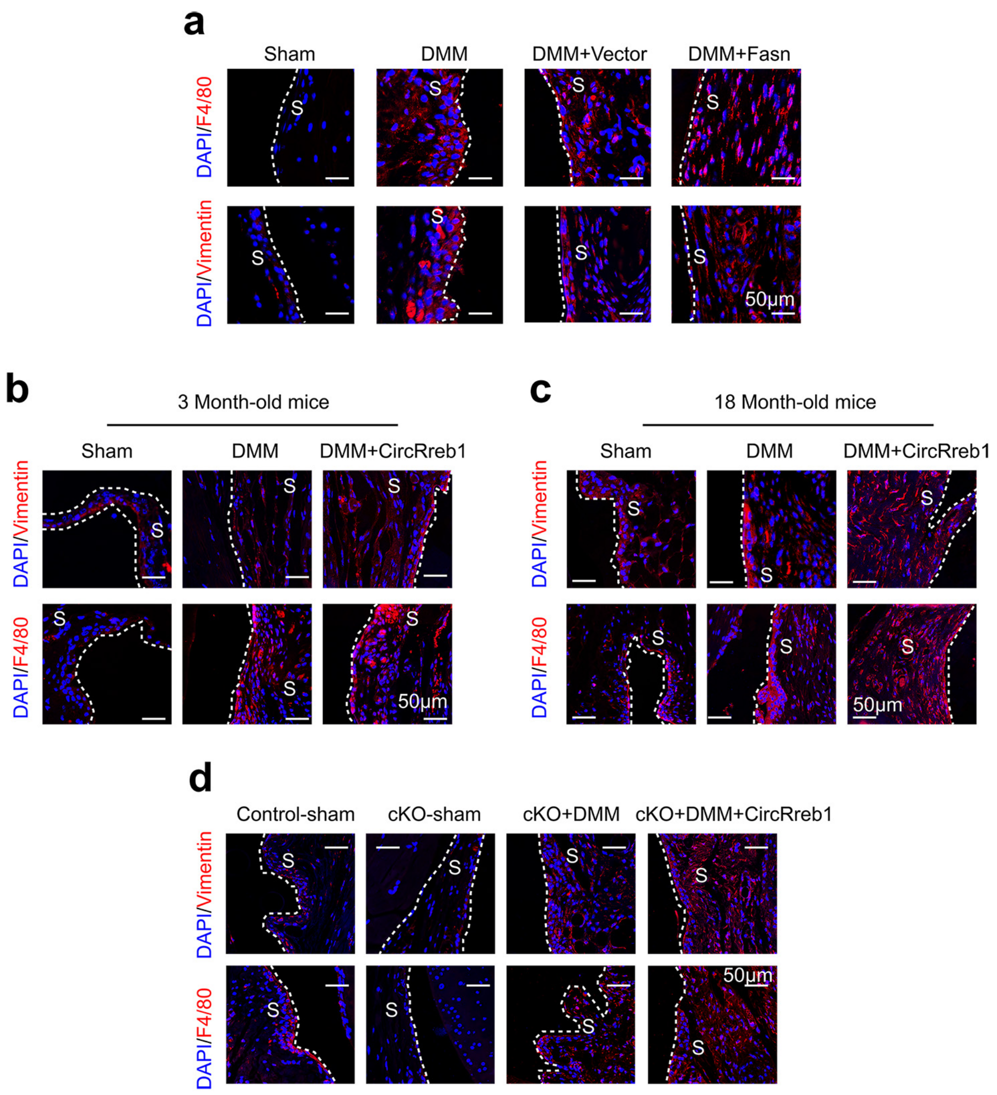

Supplementary Figure 6. a, Representative immunofluorescence images F4/80 and Vimentin in Fasn AAV based in vivo model. Images are representative of 6 independent experiments. b, c, Representative immunofluorescence images F4/80 and Vimentin in aging mice model. Images are representative of 6 independent experiments. d, Representative immunofluorescence images F4/80 and Vimentin in CircRreb1 cKO mice model. Images are representative of 6 independent experiments. All scar bar: 50 μm.

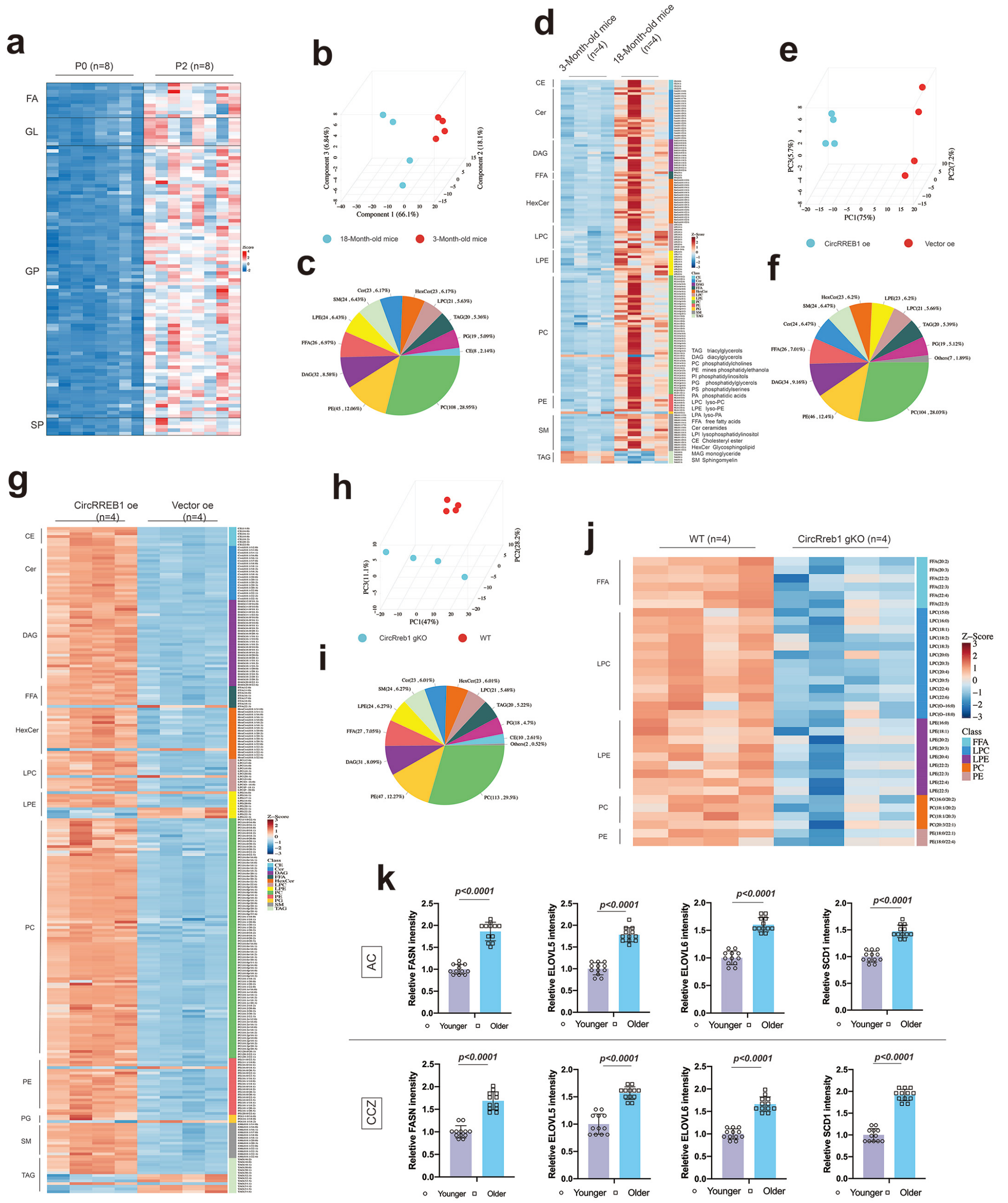

Supplementary Figure 7. a, A heat map showed the upregulated lipid types in P0 generation chondrocytes and P2 generation chondrocytes. b, Quality control of cartilage tissues from 3-month-old mice and 18-month-old mice (n=4). c, Proportion of various lipids in 3-month-old mice and 18-month-old mice. d, differentially expressed lipid types in 3-month-old mice and 18-month-old mice. e, Quality control of CircRREB1 overexpressed and vector overexpressed chondrocytes (n=4). f, Proportion of various lipids in CircRREB1 overexpressed chondrocyte and vector overexpressed chondrocyte. g, differentially expressed lipid types in CircRREB1 overexpressed chondrocyte and vector overexpressed chondrocyte. h, Quality control of cartilage

tissues from WT mice and CircRreb1 gKO mice (n=4). i, Proportion of various lipids in WT mice and CircRreb1 gKO mice. j, differentially expressed lipid types in WT mice and CircRreb1 gKO mice. k, Quantifications of the relative intensity of FASN, ELOVL5, RLOVL6, and SCD1 in articular chondrocyte (AC) and CCZ (calcified cartilage zone) between younger group and older group (n=12, biological independent samples). Two-sided Student’s *t* test is used for statistical analysis(k). Quantitative data shown as mean ± s.d. Exact p values are shown in figures. Source data are provided as a Source Data file.

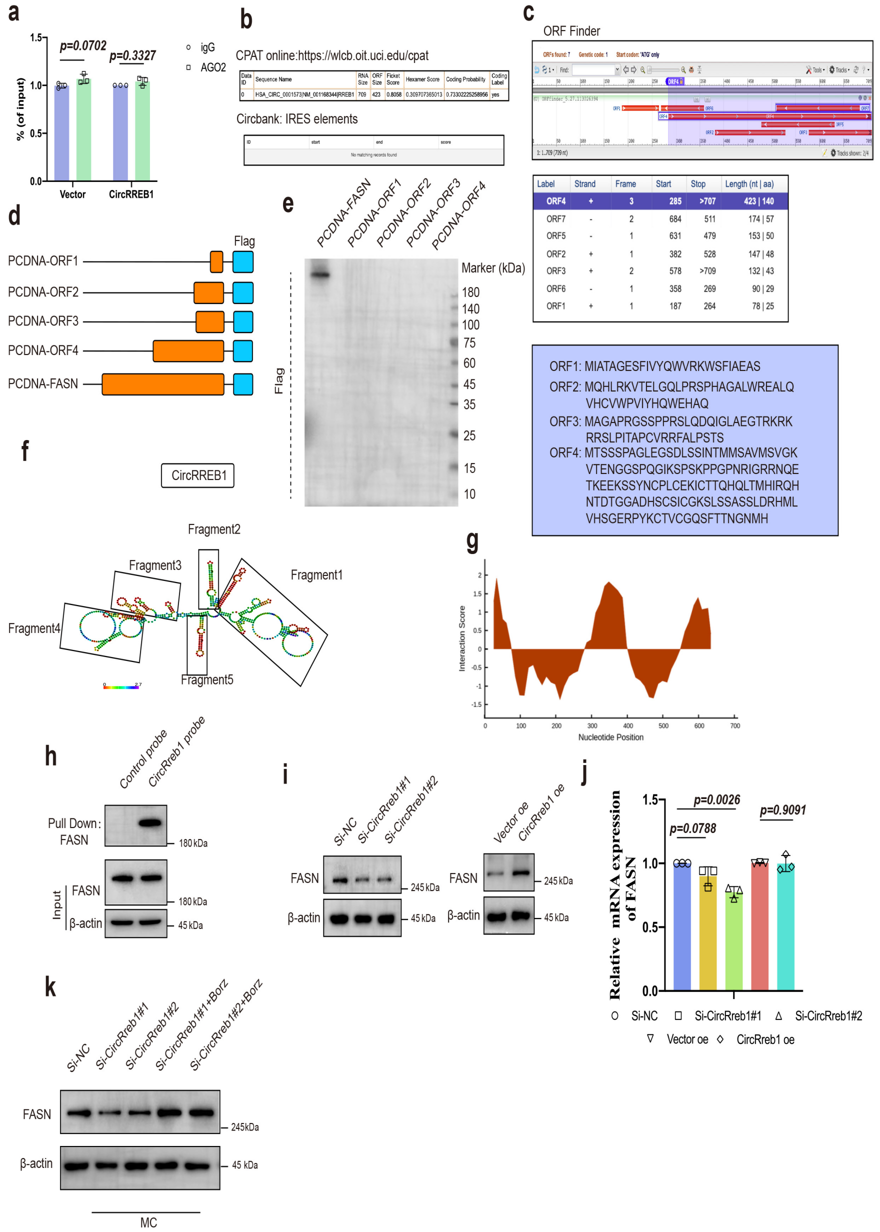

Supplementary Figure 8. a, Ago2 antibody did not combined with CircRREB1 indicated by RIP assay (n=3, biological independent samples). b, The coding ability analysis of CircRREB1. c, d, ORFs of CircRREB1 detected by ORF Finder. e, ORFs of CircREB1 did not encode proteins indicated by western blot. Blots are representative of three independent experiments. f, Fragments of CircRREB1 according to the CircRNA loop. g, the result of binding sequence of CircRREB1 with FASN predicted by CatRAPID software. h, FASN interacted with CircRreb1 in MCs after RNA pulldown indicated by western bolt. Blots are representative of three independent experiments. i, FASN expression in MCs infected with two CircRreb1 SiRNAs or CircRreb1 overexpression plasmid. Blots are representative of three independent experiments. j, FASN mRNA expression in MCs infected with two CircRreb1 SiRNAs or CircRreb1 overexpression plasmid (n=3, biological independent samples). k, the effect of Borz on FASN protein level alteration induced by CircRreb1 knockdown. Blots are representative of three independent experiments. One-way analysis of variance (ANOVA) followed by Tukey's HSD test is used for g. Two-tailed Student's *t* test is used for a (two sided). Quantitative data shown as mean ± s.d. Source data are provided as a Source Data file.

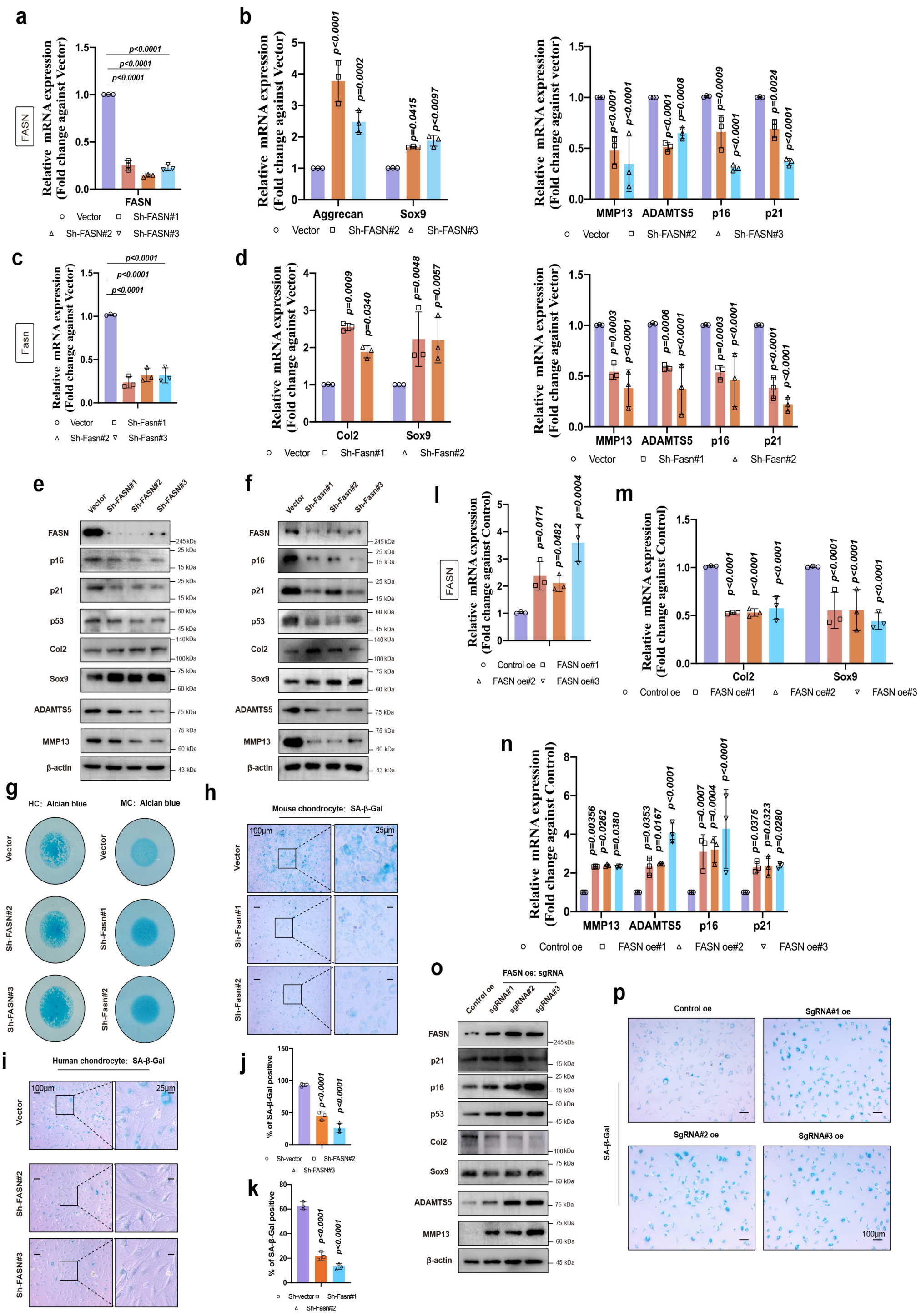

Supplementary Figure 9. The effects of FASN on ECM and senescence phenotype regulation. a, FASN knockdown efficiencies in HCs infected with three FASN ShRNAs (n=3, biological independent samples). b, *Aggrecan, Sox9, MMP13, ADAMTS5, p16*, and *P21* mRNA expression in HCs infected with Sh-FASN#2 and Sh-FASN#3 indicated by RT-qPCR (n=3, biological independent samples). c, Fasn knockdown efficiencies in MCs infected with three Fasn ShRNAs (n=3, biological independent samples). d, *Col2, Sox9, MMP13, ADAMTS5, p16*, and *P21* mRNA expression in HCs

infected with Sh-Fasn#1 and Sh-Fasn#2 indicated by RT-qPCR (n=3, biological independent samples). e, f, FASN, p16, p21, p53, Col2, Sox9, ADAMTS5, and MMP13 protein expression in HCs/MCs after FASN knockdown. g, Alcian blue staining in HCs and MCs after FASN knockdown. h, SA-β-Gal staining in MCs infected with Fasn ShRNAs. i, SA-β-Gal staining in HCs infected with FASN ShRNAs. j, k, Quantification of SA-β-Gal positive staining in HCs/MCs after FASN knockdown (n=3, biological independent samples). l, FASN overexpression efficiencies in HCs infected with three FASN sgRNAs indicated by RT-qPCR (n=3, biological independent samples). m, n, *Col2, Sox9, MMP13, ADAMTS5, p16,* and *p21* mRNA expression in HCs infected with three FASN sgRNAs indicated by RT-qPCR (n=3, biological independent samples). o, FASN, p16, p21, p53, Col2, Sox9, ADAMTS5, and MMP13 protein expression in HCs after FASN overexpression. Blots are representative of three independent experiments. p, SA-β-Gal staining in HCs after FASN overexpression. Images are representative of three independent experiments. One-way analysis of variance (ANOVA) followed by Tukey's HSD test is used for statistical analysis (a, b, c, d, j, k, l, m, and n). Quantitative data shown as mean ± s.d. Exact p values are shown in figures. Scar bar for h, i: 100 μm, amplification: 25 μm. Scar bar for p: 100 μm. Source data are provided as a Source Data file.

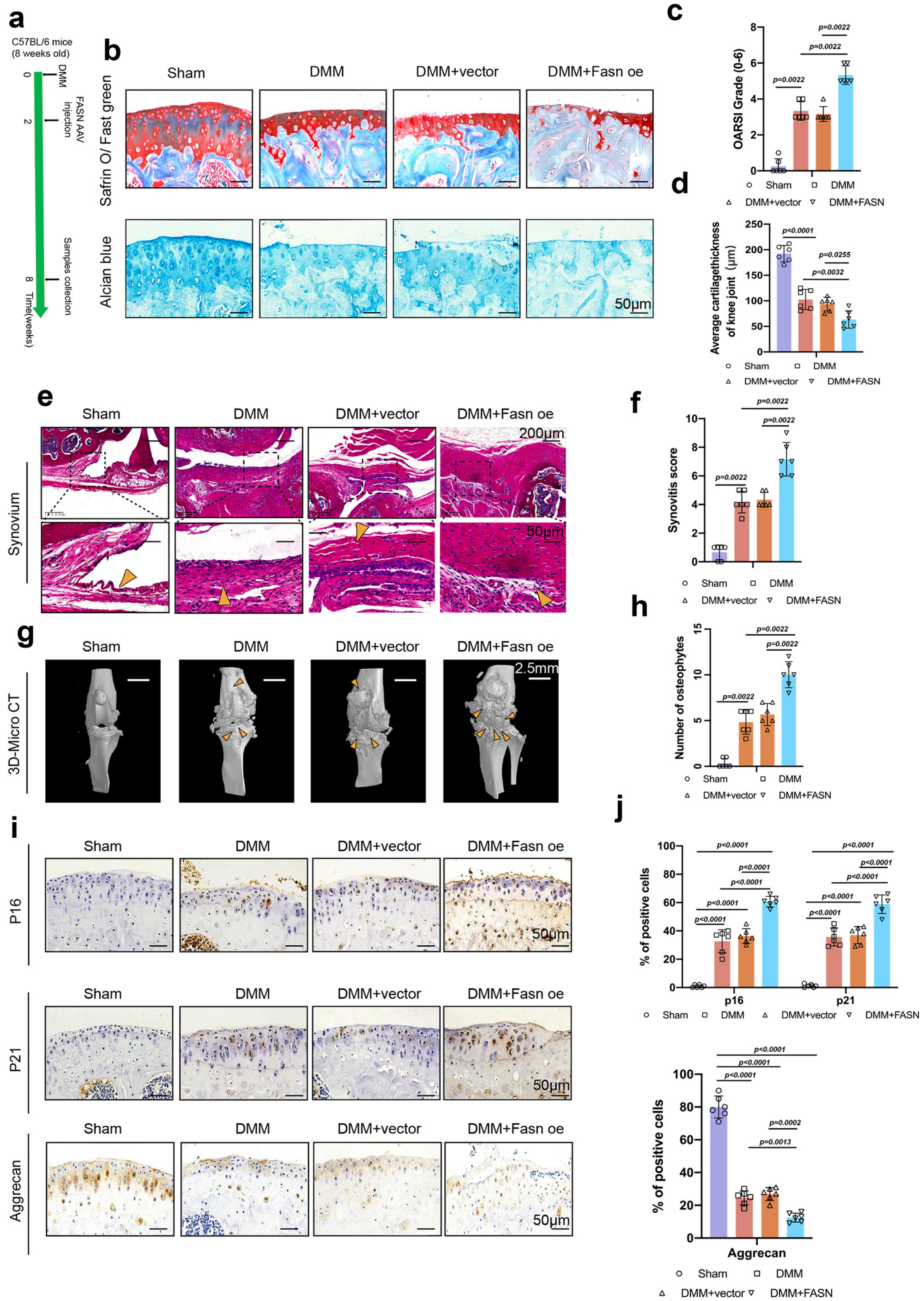

Supplementary Figure 10. Intra-articular infection of Fasn AAV aggravates age-related OA development. a, Schematic illustration of DMM operation and intra-injection of Fasn AAV in 8-week-old male wide type mice. Animal number: sham group (n=6), DMM group (n=6), DMM+ vector group (n=6), and DMM+Fasn oe group (n=6). b, SO/FG staining among four groups. c, Cartilage damage indicated by OARSI score

(n=6, biological independent samples). d, Cartilage disruption indicated by cartilage thickness (n=6, biological independent samples). e, the degrees of Synovial hyperplasia among four groups represented by H&E staining. f, OA degree indicated by synovitis score (n=6, biological independent samples). g, Images of Micro-CT analysis among four group. h, Quantification of the number of osteophytes (n=6, biological independent samples). i, Molecular detection of p16, p21, and Aggrecan expression among four groups. j, Quantifications of p16, p21, and Aggrecan among four groups (n=6, biological independent samples). The Mann-Whitney U test (two sided) is used for c, f, and h. Quantitative data shown as mean ± 95% CI. Exact p values are shown in figures. One-way analysis of variance (ANOVA) followed by Tukey's HSD test is used for d, h, j. Quantitative data shown as mean ± s.d. Exact p values are shown in figures. Scar bar for b, i: 50 μm. Scar bar for e: 200 μm, amplification: 50 μm. Scar bar for g: 2.5 mm. Source data are provided as a Source Data file.

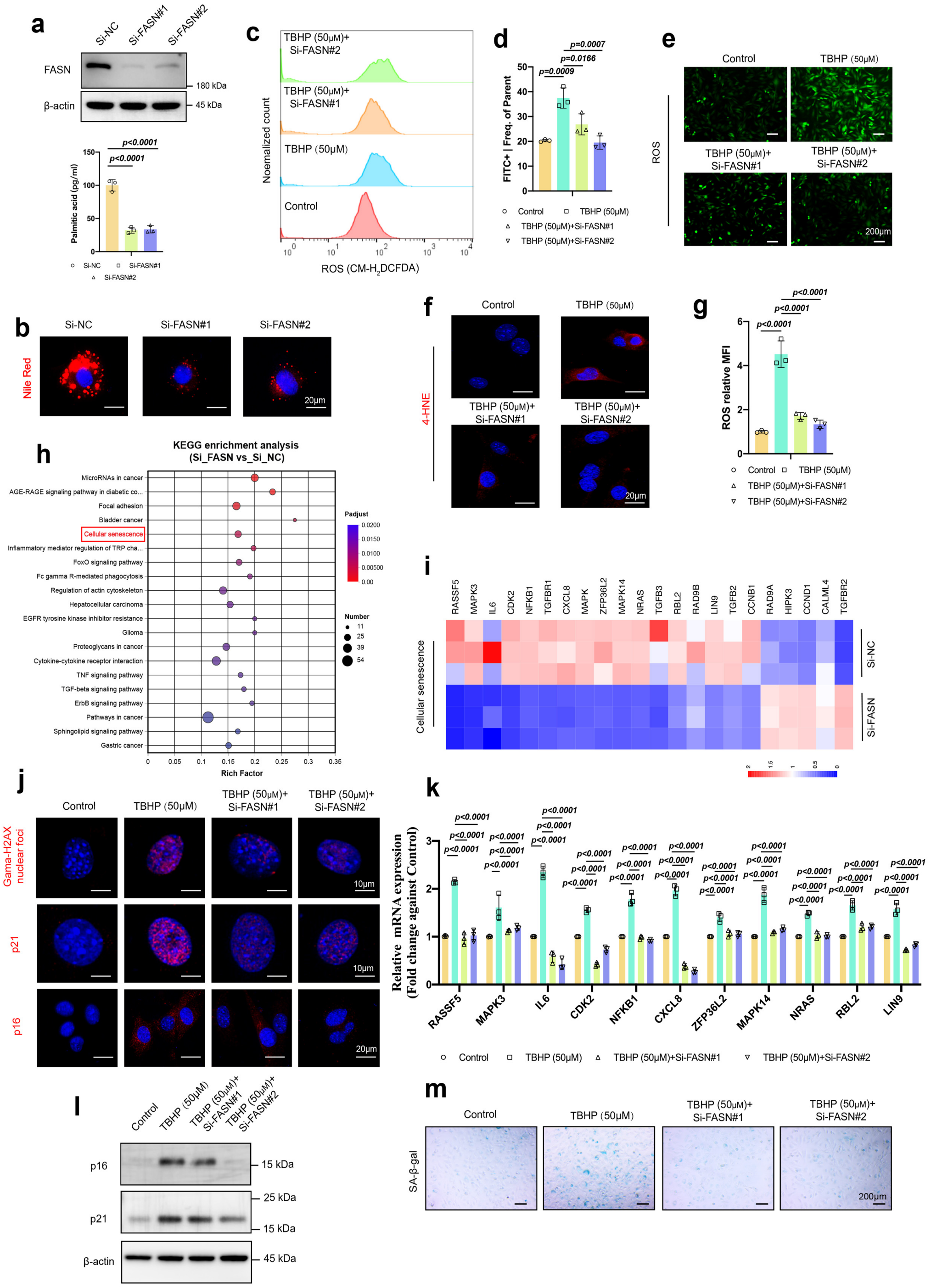

Supplementary Figure 11. FASN related lipid metabolism mediates cellular senescence by regulating ROS microenvironment. a, FASN protein expression and palmitic acid production after FASN knockdown (n=3, biological independent samples). b, lipid accumulation in chondrocyte indicated by Nile Red staining after FASN knockdown. Images are representative of three independent experiments. c, Flow cytometry of CM-H_2DCFDA. d, Quantification of FITC ROS level (n=3, biological independent samples). e, ROS expression in chondrocyte treated with TBHP alone or FASN knockdown after

TBHP treatment. f, Detection of a product of lipoid peroxidation, 4-HNE. g, Quantification of ROS intensity (n=3, biological independent samples). h, KEGG enrichment after FASN knockdown. i, A heat map of cellular senescence associated genes. j, Representative immunofluorescence images of gama-H2AX nuclear foci, p21, and p16. k, Cellular senescence associated genes expression in chondrocyte with FASN knockdown after TBHP treatment indicated by RT-qPCR (n=3, biological independent samples). l, P16 and p21 expression in in chondrocyte with FASN knockdown after TBHP treatment. Blos are representative of three independent experiments. m, SA-β-Gal staining in chondrocyte with FASN knockdown after TBHP treatment. Images are representative of three independent experiments. One-way analysis of variance (ANOVA) followed by Tukey's HSD test is used for statistical analysis (a, d, g, and k). Quantitative data shown as mean ± s.d. Exact p values are shown in figures. Scar bar for e, m: 200 μm. Scar bar for b, f: 20 μm. Scar bar for j: gama-H2AX nuclear foci, p21 (10 μm), p16 (20 μm). Source data are provided as a Source Data file.

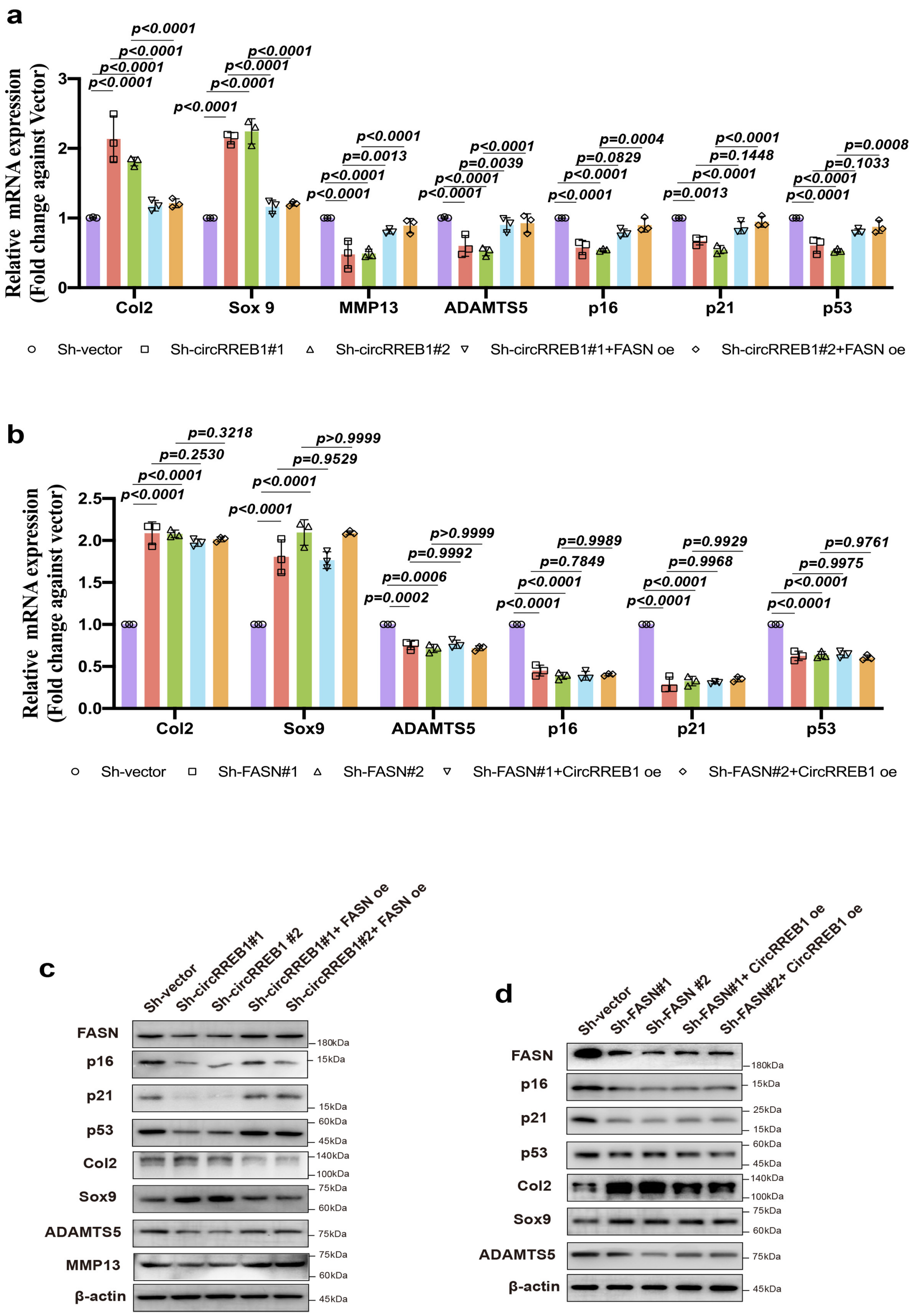

Supplementary Figure 12. a, *Col2, Sox9, MMP13, ADAMTS5, p16, p21,* and *p53* mRNA expression in HCs with CircRREB1 knockdown followed by FASN overexpression (n=3, biological independent samples). b, *Col2, Sox9, ADAMTS5, p16, p21,* and *p53* mRNA expression in HCs with FASN knockdown followed by CircRREB1 overexpression (n=3, biological independent samples). c, FASN, p16, p21, p53, Col2, Sox9, ADAMTS5, and MMP13 expression in in HCs with CircRREB1

knockdown followed by FASN overexpression. Blots are representative of three independent experiments. d, FASN, p16, p21, p53, Col2, Sox9, and ADAMTS5 expression in HCs with FASN knockdown followed by CircRREB1 overexpression. Blots are representative of three independent experiments. One-way analysis of variance (ANOVA) followed by Tukey's HSD test is used for statistical analysis (a and b). Quantitative data shown as mean ± s.d. Exact p values are shown in figures. Source data are provided as a Source Data file.

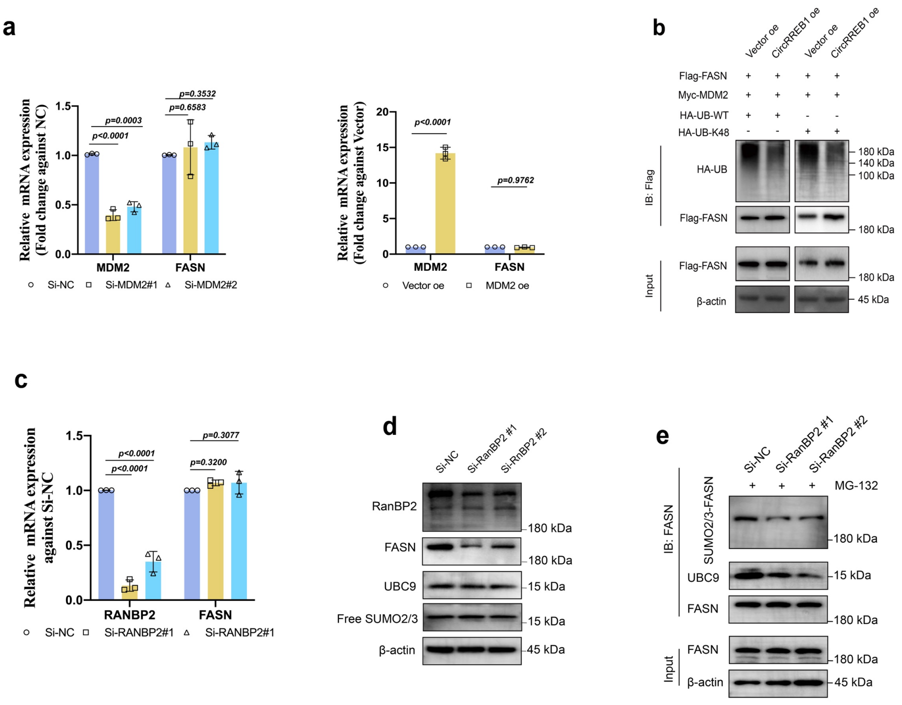

Supplementary Figure 13. a, *MDM2* knockdown or overexpression efficiency and *FASN* mRNA expression after MDM2 knockdown or overexpression indicated by RT-qPCR (n=3, biological independent samples). b, the effect of CircRREB1 on K48 type of FASN ubiquitination confirmed by Co-IP assay. c, *RanBp2* knockdown efficacy and *FASN* mRNA expression after RanBP2 knockdown indicated by RT-qPCR (n=3, biological independent samples). d, The effect of RanBP2 knockdown on FASN protein expression. Blots are representative of three independent experiments. e, The effect of RanBP2 knockdown on FASN SUMOylation. Blots are representative of three independent experiments. One-way analysis of variance (ANOVA) followed by Tukey's HSD test is used for a (left panel) and c. Two-tailed Student's t test is used for

a (right panel, two sided). Quantitative data shown as mean ± s.d. Exact p values are shown in figures. Source data are provided as a Source Data file.

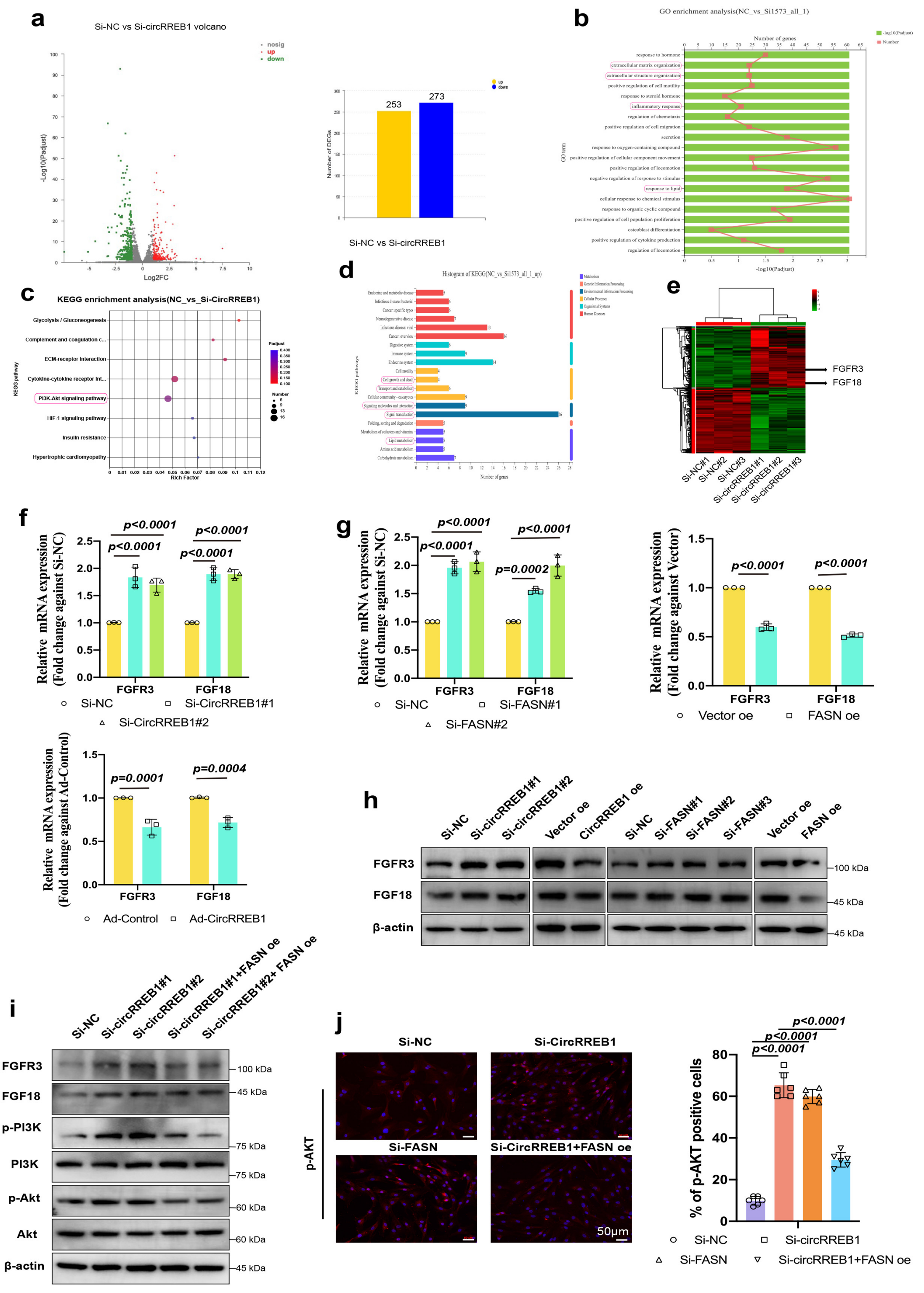

Supplementary Figure 14. CircRREB1-FASN axis regulates FGFR3 and FGF18 related PI3K-AKT signaling pathway. a, A volcano map of RNA-seq after CircRREB1 knockdown. Among differentially expressed genes, 253 of which are upregulated and 273 genes are downregulated. b, d, GO and KEGG pathway. c, PI3K-AKT signaling pathway enriched after CircRREB1 knockdown. e, A heat map with or without CircRREB1 knockdown. f, g, *FGFR3* and *FGF18* mRNA expressions in HCs infected with FASN or CircRREB1 SiRNAs and FASN plasmid or CircRREB1 adenovirus (n=3, biological independent samples). h, FGFR3 and FGF18 protein expression after CircRREB1 knockdown or overexpression and FASN knockdown or overexpression. Blots are representative of three independent experiments. i, FASN overexpression reverses the effect of CircRREB1 knockdown on PI3K-AKT signaling transduction. Blots are representative of three independent experiments. j, Immunofluorescence images of p-AKT and quantification of p-AKT in four groups. Two-sided Student's t-test is used for f (CircRREB1 overexpression) and g (FASN overexpression). One-way analysis of variance (ANOVA) followed by Tukey's HSD test is used for f (CircRREB1 knockdown), g (FASN knockdown) and j. Quantitative data shown as mean ± s.d. Exact p values are shown in figures. All scar bar: 50 μm. Source data are provided as a Source Data file.

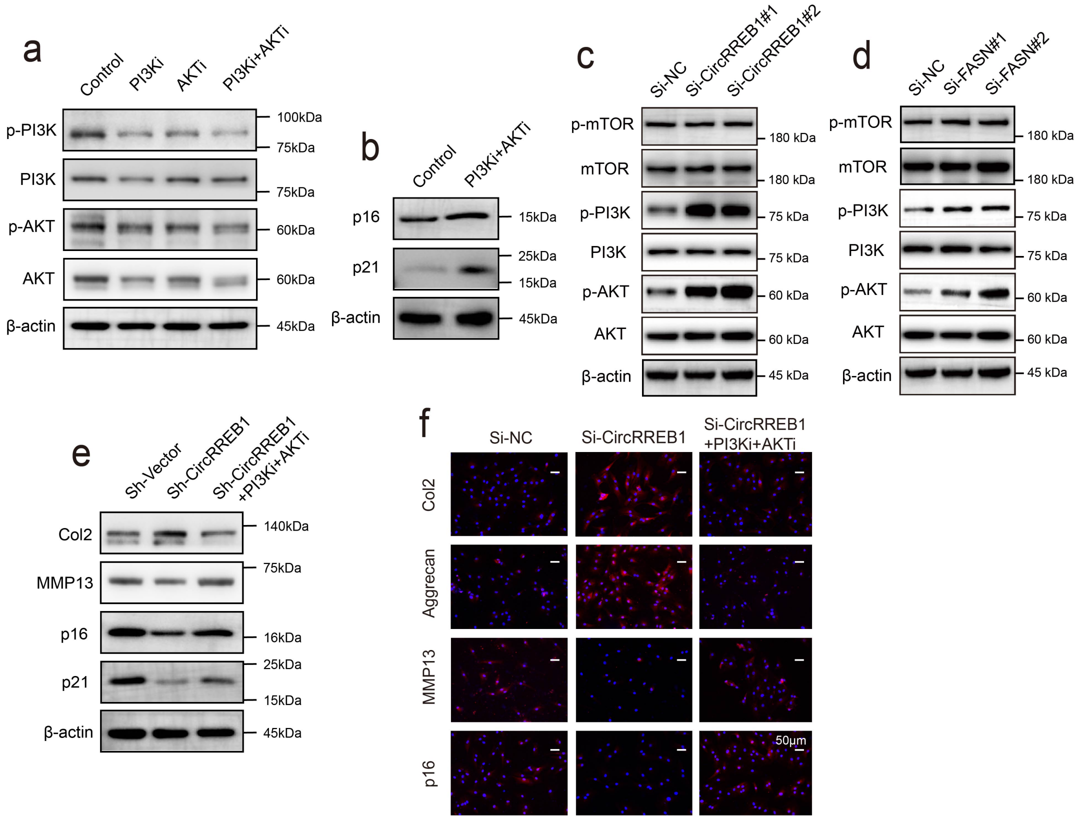

Supplementary Figure 15. PI3K inhibitor and AKT inhibitor reverses the effects of CircRREB1 knockdown. a, P-PI3K, PI3K, p-AKT, and AKT expression in HCs treated with PI3K inhibitor (PI3Ki), AKT inhibitor (AKTi) or combined treatment. Blots are representative of three independent experiments. b, p16 and p21 in HCs treated with combination of PI3K inhibitor (PI3Ki) and AKT inhibitor (AKTi). Blots are representative of three independent experiments. c, d, P-PI3K, PI3K, p-AKT, AKT, mTOR, and p-mTOR expression in HCs after CircRREB1 and FASN knockdown. Blots are representative of three independent experiments. e, PI3K added with AKTi reverse the protective effects medicated by CircRREB1 knockdown. Blots are representative of three independent experiments. f, Representative immunofluorescence images of Col2, Aggrecan, MMP13, and p16 in three groups. Images are representative of three independent experiments. All scar bar: 50 μm. Source data are provided as a Source Data file.

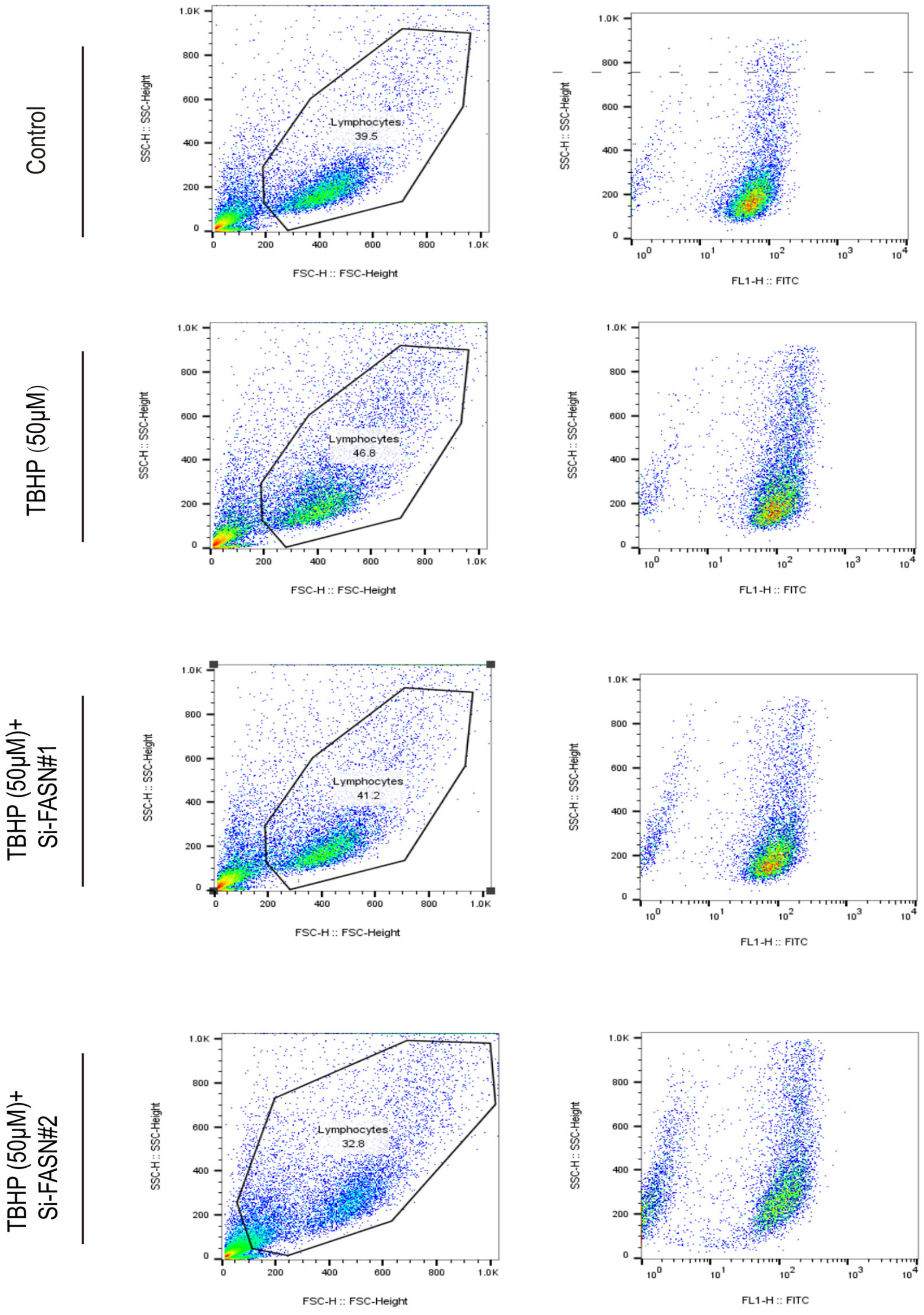

Supplementary Figure 16. Gating strategy

Representative gating strategy to evaluate ROS content, measured as 2′,7′-dichlorodihydrofluorescein diacetate (H2DCFDA) fluorescence by flow cytometry. Cell population was distinguished from cellular debris (left panel). Singlets were selected to gate DCFDA positive chondrocytes (right panel). This gating strategy corresponding to supplementary figure 11c and d.

Supplementary Tables

Supplementary Table 1. Top 15 differentially expressed circRNAs (upregulated and downregulated) in P2 generation chondrocytes and P0 generation chondrocytes. (Selection for upregulated CircRNAs: log2FC(P2/P0) >1 and Express P2 value > 300; Selection for downregulated CircRNAs: |log2FC(P2/P0) | >1 and Express P2 value < 64)

id	log2FC(P2/P0)	FDR	regulate	significant	Express_P2	Express_P0
21:29321221|29329693	4.28136117758586	≤ 0.05	Up	Yes	490.881174066667	25.234518236667
X:107840670|107854704	3.78855401571745	≤ 0.05	Up	Yes	499.704917866667	36.152122713333
16:68121987|68126610	3.69527091118186	≤ 0.05	Up	Yes	491.9865459	37.97172346
7:139715932|139717015	3.66397128122484	≤ 0.05	Up	Yes	577.415409166667	45.54443157
9:110941421|110973558	2.96153005614947	≤ 0.05	Up	Yes	882.5443391	113.29056473
11:33286413|33287511	2.95752994374638	≤ 0.05	Up	Yes	1785.631793	229.86360986667
4:186706563|186709845	2.81754365460771	≤ 0.05	Up	Yes	439.973080833333	62.40239124
6:7176655|7189322	2.80649158792688	≤ 0.05	Up	Yes	373.3015597	53.35214611

6:56851397|56900621	2.52116282482286	≤ 0.05	Up	Yes	394.498406733333	68.714347946666 7
10:31908172|31910563	2.24263891838533	≤ 0.05	Up	Yes	452.518283733333	95.608988363333 3
2:36396614|36442735	2.01216806384399	≤ 0.05	Up	Yes	507.4435067	125.787877743333 3
7:80789306|80810701	1.94125458679025	≤ 0.05	Up	Yes	426.511249866667	111.051826966667
2:54982515|54987698	1.88195169083515	≤ 0.05	Up	Yes	1032.06463533333	280.008803433333 3
8:18867674|18872733	1.8608765216803	≤ 0.05	Up	Yes	408.604264033333	112.485168823333 3
3:149846011|149921227	1.74970054742399	≤ 0.05	Up	Yes	333.037926833333	99.026295363333 3
10:7276892|7285954	-3.35777392744074	≤ 0.05	Down	Yes	6.139654073	63.033651203333 3
8:19714885|19732566	-3.1171598662681	≤ 0.05	Down	Yes	6.072360062	52.76530439
3:52412811|52414587	-2.89502668158223	≤ 0.05	Down	Yes	4.29038210366667	31.978723883333 3

7:90604218|90623543	-2.87140664680807	≤ 0.05	Down	Yes	4.43773683033333	32.537573233333 3
10:119038238|119038439	-2.86363902749853	≤ 0.05	Down	Yes	6.07284391333333	44.263913943333 3
16:14997753|14998405	-2.72248175065177	≤ 0.05	Down	Yes	9.22093870333333	60.914859553333 3
11:108176246|108177090	-2.63909402346994	≤ 0.05	Down	Yes	6.67453908833333	41.630691183333 3
10:77586317|77587871	-2.61290999585018	≤ 0.05	Down	Yes	6.28536486733333	38.501035013333 3
9:131455076|131459356	-2.55507833077876	≤ 0.05	Down	Yes	4.50503084133333	26.524807423333 3
21:37420299|37421866	-2.48558159272122	≤ 0.05	Down	Yes	10.4092487726667	58.34405274
4:153394262|153397333	-2.4490609719162	≤ 0.05	Down	Yes	6.213815288	33.975800898666 7
12:132507252|132508350	-2.43474730117682	≤ 0.05	Down	Yes	5.73017629866667	31.02538953

16:21315311|21317126	-2.42173595816713	≤ 0.05	Down	Yes	4.69773209366667	25.214752002
15:92937518|92939718	-2.42036395537787	≤ 0.05	Down	Yes	5.95594780833333	31.926079499666 7
10:121564502|121565704	-2.38576770552711	≤ 0.05	Down	Yes	4.60113954133333	24.088827166666 7

Supplementary Table 2. Top 20 CircRREB1-binding proteins identified by mass spectrometry (ranked by sum pep_score).

Number	Accession	Description	Sum PEP Score
1	Q15149	Plectin OS=Homo sapiens OX=9606 GN=PLEC PE=1 SV=3	599.031
2	Q09666	Neuroblast differentiation-associated protein AHNAK OS=Homo sapiens OX=9606 GN=AHNAK PE=1 SV=2	621.126
3	P35580	Myosin-10 OS=Homo sapiens OX=9606 GN=MYH10 PE=1 SV=3	732.753
4	A7BI36	p180/ribosome receptor OS=Homo sapiens OX=9606 GN=RRBP1 PE=2 SV=2	492.825
5	P78527	DNA-dependent protein kinase catalytic subunit OS=Homo sapiens OX=9606 GN=PRKDC PE=1 SV=3	319.091
6	Q14204	Cytoplasmic dynein 1 heavy chain 1 OS=Homo sapiens OX=9606 GN=DYNC1H1 PE=1 SV=5	295.233
7	P49792	E3 SUMO-protein ligase RanBP2 OS=Homo sapiens OX=9606 GN=RANBP2 PE=1 SV=2	291.873
8	A4QPB0	IQ motif containing GTPase activating protein 1 OS=Homo sapiens OX=9606 GN=IQGAP1 PE=1 SV=1	355.305
9	Q14690	Protein RRP5 homolog OS=Homo sapiens OX=9606 GN=PDCD11 PE=1 SV=3	217.566
10	Q9BQG0	Myb-binding protein 1A OS=Homo sapiens OX=9606 GN=MYBBP1A PE=1 SV=2	202.934
11	P49756	RNA-binding protein 25 OS=Homo sapiens OX=9606 GN=RBM25 PE=1 SV=3	322.591

12	P52272	Heterogeneous nuclear ribonucleoprotein M OS=Homo sapiens OX=9606 GN=HNRNPM PE=1 SV=3	235.743
13	P49327	Fatty acid synthase OS=Homo sapiens OX=9606 GN=FASN PE=1 SV=3	219.964
14	O75533	Splicing factor 3B subunit 1 OS=Homo sapiens OX=9606 GN=SF3B1 PE=1 SV=3	278.269
15	Q08211	ATP-dependent RNA helicase A OS=Homo sapiens OX=9606 GN=DHX9 PE=1 SV=4	227.065
16	P07814	Bifunctional glutamate/proline--tRNA ligase OS=Homo sapiens OX=9606 GN=EPRS1 PE=1 SV=5	208.798
17	D9ZGF2	Collagen, type VI, alpha 3 OS=Homo sapiens OX=9606 GN=COL6A3 PE=2 SV=1	198.353
18	Q9NR30	Nucleolar RNA helicase 2 OS=Homo sapiens OX=9606 GN=DDX21 PE=1 SV=5	283.562
19	V9HWE1	Vimentin OS=Homo sapiens OX=9606 GN=HEL113 PE=2 SV=1	248.793
20	Q9BVJ6	U3 small nucleolar RNA-associated protein 14 homolog A OS=Homo sapiens OX=9606 GN=UTP14A PE=1 SV=1	200.93

Supplementary Table 3. Top 60 Flag-FASN-binding proteins identified by mass spectrometry (ranked by LFQ intensity).

Number	Protein ID	Protein name	LFQ intensity FASN	LFQ intensity igG
1	Q53FW2	Ribose-phosphate pyrophosphokinase 3	2000200000	0
2	O95831	Apoptosis-inducing factor 1, mitochondrial	1540700000	0
3	H3BSJ9	Cytochrome b-c1 complex subunit 2, mitochondrial	1189300000	0
4	Q9UHD1	Cysteine and histidine-rich domain-containing protein 1	972840000	0
5	O00410	Importin-5	928020000	0
6	Q5U0F4	Eukaryotic translation initiation factor 3 subunit I	900280000	0
7	H0Y993	Protein DEK	896020000	0
8	O95433	Activator of 90 kDa heat shock protein ATPase homolog 1	859730000	0
9	B3KMS0	Condensin complex subunit 1	837470000	0
10	P42285	Superkiller viralicidic activity 2-like 2	813290000	0
11	F5GZS6	4F2 cell-surface antigen heavy chain	800150000	0
12	B4DSI9	Eukaryotic translation initiation factor 4 gamma 1	788770000	0
13	P38606	V-type proton ATPase catalytic subunit A	779990000	0
14	Q14008	Cytoskeleton-associated protein 5	725640000	0
15	Q9Y4L1	Hypoxia up-regulated protein 1	724530000	0
16	Q8TC62	Septin-7	723880000	0

17	B4DJ81	NADH-ubiquinone oxidoreductase 75 kDa subunit, mitochondrial	715290000	0
18	Q71UH4	DNA topoisomerase 2;DNA topoisomerase 2-beta	707810000	0
19	B2RD27	26S proteasome non-ATPase regulatory subunit 7	706730000	0
20	P07954	Fumarate hydratase, mitochondrial	675740000	0
21	Q16643	Drebrin	632390000	0
22	A6NKB8	Aminopeptidase B	626520000	0
23	B4E043	KH domain-containing, RNA-binding, signal transduction-associated protein 1	622380000	0
24	Q6IAL5	Succinyl-CoA ligase [ADP/GDP-forming] subunit alpha, mitochondrial	621360000	0
25	E9PKD5	26S protease regulatory subunit 6A	619940000	0
26	B4DKS0	Monocarboxylate transporter 1	599290000	0
27	D6W5C0	Spectrin beta chain, non-erythrocytic 1	587410000	0
28	B4DYA7	Glucose-6-phosphate 1-dehydrogenase	583550000	0
29	O00116	Alkyldihydroxyacetonephosphate synthase, peroxisomal	567200000	0
30	Q96CS3	FAS-associated factor 2	563790000	0
31	Q15738	Sterol-4-alpha-carboxylate 3-dehydrogenase, decarboxylating	550650000	0
32	Q86VX4	Structural maintenance of chromosomes protein 3	550640000	0
33	Q75L23	26S protease regulatory subunit 7	543360000	0

34	Q8N5A0	Eukaryotic translation initiation factor 5B	538470000	0
35	Q7Z6Z7	E3 ubiquitin-protein ligase HUWE1	536510000	0
36	Q53GW1	Sec1 family domain-containing protein 1	528230000	0
37	A8K048	Kinesin-like protein	522450000	0
38	Q2TAY7	WD40 repeat-containing protein SMU1	521240000	0
39	Q9UBB4	Ataxin-10	506830000	0
40	Q4G0D9	Ribosome biogenesis protein BOP1	502940000	0
41	B2R6D0	26S proteasome non-ATPase regulatory subunit 1	499440000	0
42	B3KTJ9	Cell cycle and apoptosis regulator protein 2	495400000	0
43	B4E312	TATA-binding protein-associated factor 2N	494150000	0
44	A8K6Q8	Transferrin receptor protein 1	492960000	0
45	Q0VAB1	Mitochondrial import inner membrane translocase subunit TIM50	490090000	0
46	Q53G58	Coronin-1C	487760000	0
47	E7EMV2	Neurofilament medium polypeptide	479380000	0
48	H3BR35	Eukaryotic peptide chain release factor GTP-binding subunit ERF3A	475470000	0
49	Q5I6Y5	Prelamin-A/C	474720000	0
50	O14979	Heterogeneous nuclear ribonucleoprotein D-like	472740000	0

51	B2RBP3	NEDD8-activating enzyme E1 catalytic subunit	458050000	0
52	Q59EH7	DnaJ homolog subfamily C member 7	449270000	0
53	Q86X55	Histone-arginine methyltransferase CARM1	447390000	0
54	Q9BVI4	Nucleolar complex protein 4 homologs	440650000	0
55	P49792	E3 SUMO-protein ligase RanBP2	428530000	0
56	A8K5W7	Isoleucine--tRNA ligase, mitochondrial	427300000	0
57	P17612	cAMP-dependent protein kinase catalytic subunit alpha	425130000	0
58	Q9UBE0	SUMO-activating enzyme subunit 1	423340000	0
59	B7ZM99	Monofunctional C1-tetrahydrofolate synthase, mitochondrial	417490000	0
60	Q6IBR8	Eukaryotic translation initiation factor 2 subunit 2	416350000	0

Supplementary Table 4. Differentially expressed genes related to PI3K-AKT signaling pathway after CircRREB1 knockdown

Gene_id	Gene name	Log2FC(Si1573/NC)	Significant	Regulate
ENSG00000156427	FGF18	5.426075799	yes	up
ENSG00000124253	PCK1	2.660034001	yes	up
ENSG00000068078	FGFR3	2.26032005	yes	up
ENSG00000092758	COL9A3	2.170689355	yes	up
ENSG00000144668	ITGA9	2.00779687	yes	up
ENSG00000091879	ANGPT2	1.943152397	yes	up
ENSG00000143127	ITGA10	1.59357179	yes	up
ENSG00000163235	TGFA	1.57400881	yes	up
ENSG00000113296	THBS4	1.480506754	yes	up
ENSG00000168477	TNXB	1.203756117	yes	up
ENSG00000259207	ITGB3	-1.001691826	yes	down
ENSG00000162409	PRKAA2	-1.007385624	yes	down
ENSG00000113494	PRLR	-1.081970797	yes	down
ENSG00000184371	CSF1	-1.093148067	yes	down

ENSG00000108821	COL1A1	-1.181189271	yes	down
ENSG00000029559	IBSP	-3.120891266	yes	down

Supplementary Table 5. Descriptive characteristics of human cartilage samples with different age. BMI: Body mass index.

3 knee joint tissues for CircRNA deep sequencing

Samples(n=3)	
Age (years)	57.67±2.52
Female, n(%)	3(100)
Male, n(%)	0(0)
Height (m)	1.60±0.03
Weight (kg)	60.00±3.61
BMI (kg/m2)	23.33±0.93

Clinical OA tissues for histopathological research and CircRREB1 FISH staining

Samples(n=24)	Younger, 50-65y(n=12)	Older, 70-85y(n=12)
Age (years)	59.17±4.78	78.42±4.76
Female, n(%)	6(50)	7(58.33)
Male, n(%)	6(50)	5(41.67)
Height (m)	1.67±0.09	1.63±0.07
Weight (kg)	69.85±9.58	68.75±7.77
BMI (kg/m2)	25.06±1.17	25.03±1.11

Chondrocytes from clinical OA tissues used for RT-qPCR and western blot

Samples(n=9)	
Age (years)	64.56±4.42
Female, n(%)	4(44.44)
Male, n(%)	5(55.55)
Height (m)	1.69±0.09
Weight (kg)	72.62±9.16
BMI (kg/m2)	25.41±1.09

Cartilage samples for linear regression (CircRREB1 FISH staining)

Samples(n=24)	
Age (years)	66.96±10.37
Female, n(%)	15(62.50)
Male, n(%)	9(37.50)
Height (m)	1.64±0.07
Weight (kg)	67.18±7.89
BMI (kg/m2)	24.80±1.36

Chondrocytes from clinical OA tissues used for immunofluorescence staining, SA-β-Gal staining and Alcian blue staining

Samples(n=9)	
Age (years)	67.89±3.86
Female, n(%)	4(44.44)
Male, n(%)	5(55.55)
Height (m)	1.68±0.07
Weight (kg)	71.29±8.72
BMI (kg/m2)	25.24 ±1.94

Chondrocytes from clinical OA tissues used for CircRREB1 FISH staining (P0 vs P2, Con vs Doxo)

Samples(n=12)	
Age (years)	63.08±2.02
Female, n(%)	6(50)
Male, n(%)	6(50)
Height (m)	1.67±0.08
Weight (kg)	70.69±8.91
BMI (kg/m^2)	24.83±1.34

Supplementary Table 6. Sequence of SiRNAs, ShRNA, RNA pulldown probe and FISH Probe.

SiRNAs	
Si-CircRREB1#1	GGAACATGCACAGGTTGCT
Si-CircRREB1#2	CATGCACAGGTTGCTCCGA
Si-CircRREB1#3	ACAGGTTGCTCCGACTGTG
Si-CircRreb1#1	GGAACATGCACAGGTTGTT
Si-CircRreb1#2	CATGCACAGGTTGTTGCAA
Si-CircRreb1#3	CACAGGTTGTTGCAATCCT
Si-FASN#1	GCATCAATGTCCTGCTGAA
Si-FASN#2	GCGTTGACCTGGTCTTGAA
Si-FASN#3	GCATGGCTATCTTCCTGAA
Si-Fasn#1	CCAACCGGCTCTCTTTCTT
Si-Fasn#2	GGTGGTATCCACATCTCAA
Si-Fasn#3	CCGTGGACCTTATCACTAA
Si-MDM2#1	GAGAGCAATTAGTGAGACA
Si-MDM2#2	ATCGGACTCAGGTACATCT
Si-RanBP2#1	CTAGGTGCATTGCGTGTCA
Si-RanBP2#2	GTTTCAGGGTTCTTCTAAT
Sh-CircRREB1#1	F: CCGGGGAACATGCACAGGTTGCTCTCGAGAGCAACCTGTGCATGTTCCTTTTTC<br>R: CCTTGTACGTGTCCAACGAGAGCTCTCGTTGGACACGTACAAGGAAAAAGTTAA

Sh-CircRREB1#2	F: CCGGCATGCACAGGTTGCTCCGACTCGAGTCGGAGCAACCTGTGCATGTTTTTC<br>R: GTACGTGTCCAACGAGGCTGAGCTCAGCCTCGTTGGACACGTACAAAAAGTTAA
Sh-FASN#1	F: CCGGGCATCAATGTCCTGCTGAACTCGAGTTCAGCAGGACATTGATGCTTTTTC<br>R: CGTAGTTACAGGACGACTTGAGCTCAAGTCGTCCTGTAACTACGAAAAAGTTAA
Sh-FASN#2	F: CCGGGCGTTGACCTGGTCTTGAACTCGAGTTCAAGACCAGGTCAACGCTTTTTC<br>R: CGCAACTGGACCAGAACTTGAGCTCAAGTTCTGGTCCAGTTGCGAAAAAGTTAA
Sh-FASN#3	F: CCGGGCATGGCTATCTTCCTGAACTCGAGTTCAGGAAGATAGCCATGCTTTTTC<br>R: CGTACCGATAGAAGGACTTGAGCTCAAGTCCTTCTATCGGTACGAAAAAGTTAA
Sh-Fasn#1	F: CCGGCCAACCGGCTCTCTTTCTTCTCGAGAAGAAAGAGAGCCGGTTGGTTTTTC<br>R: GGTTGGCCGAGAGAAAGAAGAGCTCTTCTTTCTCTCGGCCAACCAAAAAGTTAA

Sh-Fasn#2	F: CCGGGGTGGTATCCACATCTCAACTCGAGTTGAGATGTGGATACCACCTTTTTC<br>R: CCACCATAGGTGTAGAGTTGAGCTCAACTCTACACCTATGGTGGAAAAAGTTAA
Sh-Fasn#3	F: CCGGCCGTGGACCTTATCACTAACTCGAGTTAGTGATAAGGTCCACGGTTTTTC<br>R: GGCACCTGGAATAGTGATTGAGCTCAATCACTATTCCAGGTGCCAAAAAGTTAA
FISH probes	
Has_Circ_0001573	5‘-TCGGAGCAACCTGTGCATGTTCCCAT-3’
Mmu_Circ_0001573	5'-AAGAATGTCTTTCCTGATTACCTGTGTTT-3'
RNA pull down probe	
hsa_Circ_0001573 probe (5’- 3’)	GUCGGAGCAACCUGUGCAUGUUCCC
mmu_Circ_0001573 probe(5’- 3’)	UCUUUCCUGAUUACCUGUGUUUCUC

Supplementary Table 7. Antibodies information

Antibodies	Company	Catalog #	Application/Dilution
Col2	Bioss	bs-5881R	WB (1:1000)
Aggrecan	Abcam	ab3778	WB (1:1000)
ADAMTS5	Abcam	ab41037	WB (1:250)
MMP3	Abcam	ab52915	WB (1:2000)
MMP13	Abcam	ab39012	WB (1:5000); IF (1:200); IHC (1:100)
P16	Santa Cruz	Sc-56330	IF (1:100); IHC (1:50)
P21	Santa Cruz	Sc-6246	WB (1:200); IHC (1:50)
P53	Proteintech	60283-2-lg	WB (1:5000)
P16	Proteintech	10883-1-AP	WB (1:1000)
ADAMTS4	Abcam	Ab185722	WB (1:1000)
Beta actin	HUABIO	M1210-2	WB (1:10000)
Sox9	Abcam	Ab185966	WB (1:1000); IF (1:200)
Aggrecan	Proteintech	13880-1-AP	IHC (1:100); IF (1:250)
FASN	Abcam	Ab128870	WB (1:1000); IF (1:250); IP(1:30); IHC (1:500)
CXCL1	Proteintech	12335-1-AP	IHC (1:100)
IL-6	Abcam	Ab9324	IHC (1:100)
SCD1	Abcam	Ab236868	WB (1:1000); IHC (1:100)
ELOVL5	Affinity	DF4038	WB (1:1000); IHC (1:100)
ELOVL6	Abcam	Ab69857	WB (1:1000); IHC (1:100)
Ubiquitin	Abcam	Ab134953	WB (1:1000)
MDM2	Abcam	Ab16895	WB (1:1000)
Myc-tag	Abcam	Ab32	WB (1:200); IP (1:50)
Flag-tag	MBL	M185-3L	WB (1:10000) IP (1:100)
HA-tag	Santa Cruz	Sc-7392	WB (1:200) IP (1:50)
Acetyllysine	PTMBio	PTM-101	WB (1:1000)

SUMO2/3	PTMBio	PTM-5412	WB (1:1000)
RanBP2	Abcam	Ab64276	WB (1:1000)
UBC9	Santa Cruz	Sc-271057	WB (1:200)
FGFR3	Abcam	Ab133644	WB (1:1000)
FGF18	Proteintech	11495-1-AP	WB (1:1000); IHC (1:100)
PI3K	Abcam	Ab191606	WB (1:1000)
p-PI3K	Abcam	Ab182651	WB (1:1000); IHC (1:100)
AKT	Abcam	Ab179463	WB (1:1000)
p-AKT	Abcam	Ab192623	WB (1:1000)
p-AKT	Proteintech	66444-1-lg	IHC (1:100)
p-AKT	Santa Cruz	Sc-514032	IF (1:50)
mTOR	Abcam	Ab134903	WB (1:10000)
p-mTOR	Abcam	Ab109268	WB (1:10000)
HDAC3	Abcam	Ab32369	WB (1:5000); IP(1:25)
LC3B	Abcam	Ab192890	WB (1:2000)
SQSTM1	Abcam	Ab109012	WB (1:10000)
Alexa 488-conjugated goat anti-mouse secondary antibody	Invitrogen	A11001	IF (1:500)
Alexa 555-conjugated donkey anti-rabbit secondary antibody	Beyotime	A0453,	IF (1:500)
Goat anti-Mouse IgG	Fude Biological	FDM007	WB (1:1000)

(HRP-conjuagted)	Technology Co., Ltd		
Goat anti-Rabbit IgG (HRP-conjuagted)	Fude Biological Technology Co., Ltd	FDR007	WB (1:1000)
Anti-Mouse/Rabbit IgG for IHC	Boster Technology	SV0004	IHC (1:1000)

Supplementary Table 8. Primers utilized for qPCR.

RREB1	F: AGGTTCAGACCTATCTTCCATCA<br>R: CTGCCAATCCGATTTGGTCCT
CircRREB1(hsa)	F: CACATTCGCCAGCACAACAC<br>R: ACAGTCGGAGCAACCTGTG
Rreb1(mu)	F: AGTAATGAGCGTAGCGAGTGT<br>R: GGTCCTGGAGGTTTCATGGG
CircRREB1(mmu)	F: CCTGTGGGTGCCTGAGAAAA<br>R: TGTGTTGTGCTGACGGATGT
Human-COL2	F: ATGACAATCTGGCTCCCAAC<br>R: GAACCTGCTATTGCCCTC
Human-AGGRECAN	F: GTGCCTATCAGGACAAGGTCT<br>R: GATGCCTTTCACCACGACTTC
Human-SOX9	F: GCTCTGGAGACTTCTGAACGA<br>R: CCGTTCTTCACCGACTTCCT
Human-MMP13	F: TCGGCCACTCCTTAGGTCTT<br>R: AAGTGGCTTTTGCCGGTGTA
Human-MMP3	F: CCTACAAGGAGGCAGGCAAG<br>R: CCCGTCACCTCCAATCCAAG
Human-ADAMTS5	F: GGGCACTGGCTACTATGTGG<br>R: CGTCACAGCCAGTTCTCACA
Human-ADAMTS4	F: GTCCCATGTGCAACGTCAAG<br>R: ATGCGGCCATCTTGTCATCT
Human-p16	F: GATCCAGGTGGGTAGAAGGTC<br>R: CCCCTGCAAACTTCGTCCT
Human-p21	F: TGTCCGTCAGAACCCATGC<br>R: AAAGTCGAAGTTCCATCGCTC
Human-p53	F: CAGCACATGACGGAGGTTGT<br>R: TCATCCAAATACTCCACACGC
Human-FASN	F: AAGGACCTGTCTAGGTTTGATGC<br>R: TGGCTTCATAGGTGACTTCCA
Human-ELOVL5	AGTGGTGTATAACCTTGGACTCA

	ACCAGAGGACACGGATAATCTTC
Human-ELOVL6	AACGAGCAAAGTTTGAACTGAGG<br>TCGAAGAGCACCGAATATACTGA
Human-SCD1	TCTAGCTCCTATACCACCACCA<br>TCGTCTCCAACTTATCTCCTCC
Human-LMNB1	AAGCATGAAACGCGCTTGG<br>AGTTTGGCATGGTAAGTCTGC
Human-FGFR3	CCCAAATGGGAGCTGTCTCG<br>CCCGGTCCTTGTCAATGCC
Human-FGF18	CACCAGCAAGGAGTGTGTGTT<br>CACCGTCGTGTACTTGAAGGG
Human-MDM2	GAATCATCGGACTCAGGTACATC<br>TCTGTCTCACTAATTGCTCTCCT
Human-RanBP2	AAACCTCCGATTGCAGCTCAT<br>GGCAAAGATGGCCTTAATCCT
Human-actin	F: AGAGCTACGAGCTGCCTGAC<br>R: AGCACTGTGTTGGCGTACAG
Mouse-Aggrecan	F: CACTGTCAAAGCACCATGCC<br>R: TAGGCTGGCTCCCATTCAGT
Mouse-Col2	F: TGGCTTAGGGCAGAGAGAGA<br>R: CGTCGTGCTGTCTCAAGGT
Mouse-Sox9	F: TAATTCCCCAGGCTCTTGGAT<br>R: GCAGCCGGGATTTAAGGCTC
Mouse-Mmp3	F: ACTGTGTCCCAAGGAGAGGAG<br>R: AAACCATCTACACAGTTCAGACAC
Mouse-Mmp13	F: CAAGCAGTTCCAAAGGCTACA<br>R: TAGGGCTGGGTCACACTTCT
Mouse-Adamts5	F: ATGCAGCCATCCTGTTCACC<br>R: AAGGCCAAGTAGATGCCCAATTT
Mouse-Adamts4	F: TTGTTCTCCCAGTCACCCTCC<br>R: AGCCTGGGACTAAAGATAGGCA
Mouse-p16	F: TGTTGAGGCTAGAGAGGATCTTG

	R: CGAATCTGCACCGTAGTTGAGC
Mouse-p21	F: TCGCTGTCTTGCACTCTGGTGT<br>R: CCAATCTGCGCTTGGAGTGATAG
Mouse-Fasn	F: GGAGGTGGTGATAGCCGGTAT<br>R: TGGGTAATCCATAGAGCCCAG
Mouse-Lmnb1	F: CCGGCCTCAAGGCTCTCTA<br>R: TGCCGCCTCATACTCTCGAA
Mouse-actin	F: GAGACCTTCAACACCCCAGC<br>R: ATGTCACGCACGATTTCCC
CircBACH1	F: AGAAATTGAGAAGCTGGTTGATGA<br>R: TGACATCGCACAGCACATCT
CircMID2	F: TATGCAAACTGGTGGGTCGT<br>R: CTGGGCTTTCACCTCAACCT
CircNFATC3	F: ACCTCCACTAGACTGGCCTT<br>R: GGCTCAAGATCTTCACAACAGG
CircHIPK2	F: GTCCAGATATTACAGGTATGGCCT<br>R: CAGGGGGATGTTCTTGCTCT
CircHIPK3	F: TCGGTACTACAGGTATGGCCT<br>R: ACACAACTGCTTGGCTCTACT
CircLPAR1	F: TGGTCATTGTGCTTGGTGGA<br>R: TGTGTACCTGATGCTGTAGGTG
CircFAT1	F: GTGAAGAGACAGATTCCCGAC<br>R: CTGTTCAAGTCGTTGGCTGC
CircDST	F: GATTGTGTGAACTTGGGCCAT<br>R: TTGGCAGACGATGGCTACTG
CircARHGAP12	F: TGGTCATTTTCCCGGTCCAG<br>R: GCTTAGAACAAGGAGATCTACACCA
CircSFMBT2	F: TCCCTTATTGATGCTGCCAAA<br>R: TTTCCCTCGCAGAGGACCTT
CircPHF7	F: GTTTGCTGGCTATGCCTTCG<br>R: ACAGGCTCTTCCAGAAGACAG
CircKCNMA1	F: TGCCAAGCAGTTTTTGCAGG

	R: GCCTCTCTGCCAGCTTCTTAAA
CircCSGALNACT1	F: GTGAAAGTCCCATGAAGACACA<br>R: TATGCACCAGGATCAGTCGC
CircEIF3A	F: CCACTCCAGGGCAGATAGCA<br>R: GCATCTTCACTGCCTCCTTCA
CircPRRC2B	F: GAAAGGCGCCAGACCCTC<br>R: TGCCGTCCTTCACAACTTCTT
CircPDXC1	F: CCACTCCAGGGCAGATAGCA<br>R: GCATCTTCACTGCCTCCTTCA
CircDYRK1A	F: AGTTTGACCTTTTGGTGCAGG<br>R: AAGAGTCCAGCGGCAAAACTAT
CircFBRSL1	F: CGATGGGGCGAGAAAGGTC<br>R: CGCGTCAAGTTTAGGGTCCA
CircNPAT	F: CCAATCCCAGTATAGTCTTAGCAG<br>R: AAGAGTGCCTGAAATGCTGGG

Supplementary Table 9. OARSI evaluation

Grade	Osteoarthritic damage
0	Normal
0.5	Loss of Safranin-O without structural changes
1	Small fibrillations without loss of cartilage
2	Vertical clefts down to the layer immediately below the superficial layer and some loss of surface lamina
3	Verical clefts/erosion to the calcified cartilage extending to <25% of the articular surface
4	Verical clefts/erosion to the calcified cartilage extending to 25-50% of the articular surface
5	Verical clefts/erosion to the calcified cartilage extending to 50-75% of the articular surface
6	Verical clefts/erosion to the calcified cartilage extending to >75% of the articular surface

Supplementary Table 10. A scheme for synovitis evaluation

Enlargement of the synovial lining cell layer	
0	The lining cells form one layer
1	The lining cells form 2-3 layers
2	The lining cells form 4-5 layers, few multinucleated cells might occur
3	The lining cells form more than 5 layers, the lining might be ulcerated and multinucleated cells might occur
Density of the resident cells	
0	The synovial stroma shows normal cellularity
1	The cellularity is slightly increased
2	The cellularity is moderately increased, multinucleated cells might occur
3	The cellularity is greatly increased, multinucleated giant cells, pannus formation and rheumatoid granulomas might occur
Inflammatory infiltrate	
0	No inflammatory infiltrate
1	Few mostly perivascular situated lymphocytes or plasma cells
2	Numerous lymphocytes or plasma cells, sometimes forming follicle-like aggregates
3	Dense band-like inflammatory infiltrate or numerous large follicle-like aggregates
Sum 0 or 1	No synovitis
Sum 2-4	Low-grade synovitis

Sum 5-9	High-grade synovitis
