# Supplementary material for: CircRREB1 mediates lipid metabolism related senescent phenotypes in chondrocytes through FASN post-translational modifications
Source: Nat Commun. 2023 Aug 28;14:5242. doi: 10.1038/s41467-023-40975-7 (PMC10462713; doi:10.1038/s41467-023-40975-7)
Supplement: Supplementary file 3 — Description of Additional Supplementary Files [file 41467_2023_40975_MOESM3_ESM.pdf]

**Description of Additional Supplementary Files**

File Name: Supplementary Data 1

Description: All metabolites detected in P0 generation chondrocytes and P2 generation chondrocytes.

File Name: Supplementary Data 2

Description: All metabolites detected in cartilage tissues of 3-month-old mice and 18-month-old mice.

File Name: Supplementary Data 3

Description: All metabolites detected in chondrocytes infected with vector or CircRreb1 adenovirus.

File Name: Supplementary Data 4

Description: All metabolites detected in cartilage tissues of wide type mice and CircRreb1 gKO mice.
