# Supplementary material for: CircRREB1 mediates lipid metabolism related senescent phenotypes in chondrocytes through FASN post-translational modifications
Source: Nat Commun. 2023 Aug 28;14:5242. doi: 10.1038/s41467-023-40975-7 (PMC10462713; doi:10.1038/s41467-023-40975-7)
Supplement: Supplementary file 8 — Reporting Summary [file 41467_2023_40975_MOESM8_ESM.pdf]

nature portfolio

Corresponding author(s): Shunwu Fan, Xiangqian Fang, Shuying Shen

Last updated by author(s): Jul 15, 2023

Reporting Summary

Nature Portfolio wishes to improve the reproducibility of the work that we publish. This form provides structure for consistency and transparency in reporting. For further information on Nature Portfolio policies, see our Editorial Policies and the Editorial Policy Checklist.

Statistics

For all statistical analyses, confirm that the following items are present in the figure legend, table legend, main text, or Methods section.

n/a	Confirmed	
☐	☒	The exact sample size (*n*) for each experimental group/condition, given as a discrete number and unit of measurement
☐	☒	A statement on whether measurements were taken from distinct samples or whether the same sample was measured repeatedly
☐	☒	The statistical test(s) used AND whether they are one- or two-sided<br>*Only common tests should be described solely by name; describe more complex techniques in the Methods section.*
☒	☐	A description of all covariates tested
☒	☐	A description of any assumptions or corrections, such as tests of normality and adjustment for multiple comparisons
☐	☒	A full description of the statistical parameters including central tendency (e.g. means) or other basic estimates (e.g. regression coefficient) AND variation (e.g. standard deviation) or associated estimates of uncertainty (e.g. confidence intervals)
☒	☐	For null hypothesis testing, the test statistic (e.g. *F*, *t*, *r*) with confidence intervals, effect sizes, degrees of freedom and *P* value noted<br>*Give P values as exact values whenever suitable.*
☒	☐	For Bayesian analysis, information on the choice of priors and Markov chain Monte Carlo settings
☒	☐	For hierarchical and complex designs, identification of the appropriate level for tests and full reporting of outcomes
☒	☐	Estimates of effect sizes (e.g. Cohen's *d*, Pearson's *r*), indicating how they were calculated

Our web collection on statistics for biologists contains articles on many of the points above.

Software and code

Policy information about availability of computer code

Data collection	1.The micro-CT data were collected by using a u-QCT system SkyScan1176 (Bruker, Kartuizersweg, Belgium).<br>2.RT-qPCR: Applied Biosystems 7500 Sequencing Detection System (version. 2.3; Foster City, CA, USA)<br>3.Western blot analysis: Amersham Imager 600 (USA) and BIO-RAN ChemiDoc XRS+system (version.3.4.2)<br>4.Immunofluorescence analysis: confocal microscope(Lecia, Germany)<br>5.Immunohistochemistry collection: KF-PRO Digital pathological scanner (Zhejiang, China)
Data analysis	Data analysis was performed by using GraphPad Prism software (version 8.0). Images were analyzed by Image J software (version v.1.51a, National Institutes of Health, MD, USA). Flow cytometer data were analyzed by BD FACSDiva (version9.0)

For manuscripts utilizing custom algorithms or software that are central to the research but not yet described in published literature, software must be made available to editors and reviewers. We strongly encourage code deposition in a community repository (e.g. GitHub). See the Nature Portfolio guidelines for submitting code & software for further information.

Data

Policy information about availability of data

All manuscripts must include a data availability statement. This statement should provide the following information, where applicable:

- Accession codes, unique identifiers, or web links for publicly available datasets
- A description of any restrictions on data availability
- For clinical datasets or third party data, please ensure that the statement adheres to our policy

The authors declare that the data supporting the findings of this study are available with the paper and its Supplementary information files. A reporting summary for this article is available as Supplementary Information file. CircRREB1 was recorded in Circbank database (http://www.circbank.cn). The Circular RNA deeo sequencing data generated in this study has been deposited in the GEO database under accession code GSE236856[https://www.ncbi.nlm.nih.gov/geo/query/acc.cgi?acc=GSE236856].The mRNA sequencing in human chondrocyte with or without CircRREB1 knockdown has been deposited in the GEO database under accession code GSE237298 [https://www.ncbi.nlm.nih.gov/geo/query/acc.cgi?acc=GSE237298]. The mRNA sequencing in human chondrocyte with or without FASN knockdown has been deposited in the GEO database under accession code GSE237011 [https://www.ncbi.nlm.nih.gov/geo/query/acc.cgi?acc=GSE237011]. Source data are provided with this paper.

Human research participants

Policy information about studies involving human research participants and Sex and Gender in Research.

Reporting on sex and gender	Human OA samples were collected from 81 OA patients (female 45 male 36) who received total knee joint replacement.
Population characteristics	Human OA cartilage samples were collected from patients undergoing total knee replacement (TKA). Participants (50–65 years old) were included into the younger group, and participants (70–85 years old) were included into the older group. The information of human OA cartilage samples were listed in the manuscript.
Recruitment	Patients with a osteoarthritis undergoing arthroplasty were recruited for providing the cartilage tissue. The collection of human cartilage samples was approved by the Ethics Committee of the Sir Run Run Shaw Hospital (Zhejiang, China), and the methods followed the guidelines set by the Declaration of Helsinki. Written informed consent was obtained from participants. Descriptive characteristics of human cartilage samples like BMI, height, and body weight was listed in Supplementary Table S5. The patient has no history of drug OA treatment, including steroid or non-steroidal anti-inflammatory drug (NSAID) treatment, within 3 years before total knee replacement surgery. Recruitment bias is unlikely to impact this study because patient comparisons are not performed and analysis of demographic or clinical covariates on cellular properties are not performed.
Ethics oversight	This study was performed with the approval of the Human Ethics Committee of Sir Run Run Shaw hospital of Zhejiang University

Note that full information on the approval of the study protocol must also be provided in the manuscript.

Field-specific reporting

Please select the one below that is the best fit for your research. If you are not sure, read the appropriate sections before making your selection.

☒ Life sciences ☐ Behavioural & social sciences ☐ Ecological, evolutionary & environmental sciences

For a reference copy of the document with all sections, see nature.com/documents/nr-reporting-summary-flat.pdf

Life sciences study design

All studies must disclose on these points even when the disclosure is negative.

Sample size	Sample size was chosen to ensure an adequate statistical power. For in vitro study, 3 biological replicates were used. The number of animal were described in the manuscript.
Data exclusions	No data were excluded from this study.
Replication	All experimental findings were reproduced in dependently as least three times. The number of biologically independent samples, mice per group, or human specimens were listed in the figure legend. Quantitative data shown as mean ± s.d.
Randomization	Human or mouse chondrocytes were randomly assigned to each experimental group. Animals were also randomly assigned to each experimental group
Blinding	OARSI grade, Synovitis score evaluation, cartilage thickness evaluation were performed by individuals who were blinded to the specific conditions of the experimental group.

Reporting for specific materials, systems and methods

We require information from authors about some types of materials, experimental systems and methods used in many studies. Here, indicate whether each material, system or method listed is relevant to your study. If you are not sure if a list item applies to your research, read the appropriate section before selecting a response.

Materials & experimental systems

n/a	Involved in the study
☐	☒ Antibodies
☐	☒ Eukaryotic cell lines
☒	☐ Palaeontology and archaeology
☐	☒ Animals and other organisms
☒	☐ Clinical data
☒	☐ Dual use research of concern

Methods

n/a	Involved in the study
☒	☐ ChIP-seq
☐	☒ Flow cytometry
☒	☐ MRI-based neuroimaging

Antibodies

Antibodies used

Primary antibodies:
Col2: Bioss, China, bs-5881R, Rabbit, WB (1:1000)
Aggrecan: Abcam, CA, USA, ab3778, Mouse, WB (1:1000)
ADAMTS5: Abcam, CA, USA, ab41037, Rabbit, WB (1:250)
MMP3: Abcam, CA, USA, ab52915, Rabbit, WB (1:2000)
MMP13: Abcam, CA, USA, ab39012, Rabbit, WB (1:5000); IF (1:200); IHC (1:100)
P16: Santa Cruz, USA, Sc-56330, Mouse, IF (1:100); IHC (1:50)
P21: Santa Cruz, USA, Sc-6246, Mouse, WB (1:200); IHC (1:50)
P53: Proteintech, China, 60283-2-lg, Rabbit, WB (1:5000)
P16: Proteintech, China, 10883-1-AP, Rabbit, WB (1:1000)
ADAMTS4: Abcam, CA, USA, Ab185722, Rabbit, WB (1:1000)
Beta actin: HUABIO, China, M1210-2, Mouse, WB (1:10000)
Sox9: Abcam, CA, USA, Ab185966, Rabbit, WB (1:1000); IF (1:200)
Aggrecan: Proteintech, 1China, 3880-1-AP, Rabbit, IHC (1:100); IF (1:250)
FASN: Abcam, CA, USA, Ab128870, Rabbit, WB (1:1000); IF (1:250); IP(1:30); IHC (1:500)
CXCL1: Proteintech, China, 12335-1-AP, Rabbit, IHC (1:100)
IL-6: Abcam, CA, USA, Ab9324, Rabbit, IHC (1:100)
SCD1: Abcam, CA, USA, Ab236868, Rabbit, WB (1:1000); IHC (1:100)
ELOVL5: Affinity, China, DF4038, Rabbit, WB (1:1000); IHC (1:100)
ELOVL6: Abcam, CA, USA, Ab69857, Rabbit, WB (1:1000); IHC (1:100)
Ubiquitin: Abcam, CA, USA, Ab134953, Rabbit, WB (1:1000)
MDM2: Abcam, Ab16895, Ab16895, Mouse, WB (1:1000)
Myc-tag: Abcam, Ab16895, Ab32, Mouse, WB (1:200); IP (1:50)
Flag-tag: MBL, USA, M185-3L, Mouse, WB (1:10000) IP (1:100)
HA-tag: Santa Cruz, USA, Sc-7392, Mouse, WB (1:200) IP (1:50)
Acetyllysine: PTMBio, China, PTM-101, Mouse, WB (1:1000)
SUMO2/3: PTMBio, China, PTM-5412, Mouse, WB (1:1000)
RanBP2: Abcam, CA, USA, Ab64276, Rabbit, WB (1:1000)
UBC9: Santa Cruz, USA, Sc-271057, Mouse, WB (1:200)
FGFR3: Abcam, CA, USA, Ab133644, Rabbit, WB (1:1000)
FGF18: Proteintech, China, 11495-1-AP, Rabbit, WB (1:1000); IHC (1:100)
PI3K: Abcam, CA, USA, Ab191606, Rabbit, WB (1:1000)
p-PI3K: Abcam, CA, USA, Ab182651, Rabbit, WB (1:1000); IHC (1:100)
AKT: Abcam, CA, USA, Ab179463, Rabbit, WB (1:1000)
p-AKT: Abcam, CA, USA, Ab192623, Rabbit, WB (1:1000)
p-AKT: Proteintech, China, 66444-1-lg, Rabbit, IHC (1:100)
p-AKT: Santa Cruz, USA, Sc-514032, Mouse, IF (1:50)
mTOR: Abcam, CA, USA, Ab134903, Rabbit, WB (1:10000)
p-mTOR: Abcam, CA, USA, Ab109268, Rabbit, WB (1:10000)
HDAC3: Abcam, CA, USA, Ab32369, Rabbit, WB (1:5000); IP(1:25)
LC3B: Abcam, CA, USA, Ab192890, Rabbit, WB (1:2000)
SQSTM1: Abcam, CA, USA, Ab109012, Rabbit, WB (1:10000)

Secondary antibodies:

Alexa 488-conjugated goat anti-mouse secondary antibody: Invitrogen, USA, Cat. no. A11001, IF(1:500)
Alexa 555-conjugated donkey anti-rabbit secondary antibody: Beyotime, China, Cat. no. A0453, IF (1:500)
Goat anti-Mouse IgG: Fude Biological Technology Co., Ltd, China, Cat. no. FDM007, WB (1:1000, HRP-conjuagted)
Goat anti-Rabbit IgG: Fude Biological Technology Co., Ltd, China, Cat. no. FDR007, WB (1:1000, HRP-conjuagted)
Anti Mouse/Rabbit IgG: Boster Technology, Cat. no. SV0004, IHC (1:1000, HRP-conjuagted)

Validation	All antibodies used in this study were validated by the supplier as follows: Col2 (http://www.bioss.com.cn/prolook_03.asp?id=AF08169606009737&pro37=1), Aggrecan (https://www.abcam.cn/products/primary-antibodies/aggrecan-antibody-6-b-4-ab3778.html), ADAMST5 (https://www.abcam.cn/products/primary-antibodies/adamts5-antibody-ab41037.html), MMP3 (https://www.abcam.cn/products/primary-antibodies/mmp3-antibody-ep1186y-ab52915.html), MMP13 (https://www.abcam.cn/products/primary-antibodies/mmp13-antibody-ab39012.html), p16 (https://www.scbt.com/p/p16-antibody-jc8?requestFrom=search), p21 (https://www.scbt.com/p/p21-antibody-f-5?requestFrom=search), p53 (https://www.ptgcn.com/products/P53-Antibody-60283-2-Ig.htm), p16 (https://www.ptgcn.com/products/P16,P19-Antibody-10883-1-AP.htm), ADAMTS4 (https://www.abcam.cn/products/primary-antibodies/adamts4-antibody-ab185722.html), beta-actin (http://www.huabio.cn/product/Beta-actin-antibody-M1210-2), Sox9 (https://www.abcam.cn/products/primary-antibodies/sox9-antibody-epr14335-78-ab185966.html), Aggrecan (https://www.ptgcn.com/products/ACAN-Antibody-13880-1-AP.htm), FASN (https://www.abcam.cn/products/primary-antibodies/fatty-acid-synthase-antibody-epr7466-ab128870.html), CXCL1 (https://www.ptgcn.com/products/CXCL1-Antibody-12335-1-AP.htm), IL-6 (https://www.abcam.cn/products/primary-antibodies/il-6-antibody-12-2b11-2g10-ab9324.html), SCD1 (https://www.abcam.cn/products/primary-antibodies/scd1-antibody-epr21963-ab236868.html), ELOVL5 (https://www.affbiotech.cn/goods-3106-DF4038-ELOVL5_Antibody.html), ELOVL6 (https://www.abcam.cn/products/primary-antibodies/elovl6lce-antibody-ab69857.html), Ubiquitin (https://www.abcam.cn/products/primary-antibodies/ubiquitin-antibody-epr8830-ab134953.html), MDM2 (https://www.abcam.cn/products/primary-antibodies/mdm2-antibody-2a10-ab16895.html), Myc-tag (https://www.abcam.cn/products/primary-antibodies/myc-tag-antibody-9e10-ab32.html), Flag-tag (http://www.ny-bio.com/nd.jsp?id=582), HA-tag (https://www.scbt.com/p/ha-probe-antibody-f-7?requestFrom=search), Acetyllysine (http://www.ptm-biolab.com.cn/productDetail.html?id=4610), SUMO2/3 (http://www.ptm-biolab.com.cn/productDetail.html?id=5141), RanBP2 (https://www.abcam.cn/products/primary-antibodies/ranbp2-antibody-ab64276.html), UBC9 (https://www.scbt.com/p/ubc9-antibody-c-12?requestFrom=search), FGFR3 (https://www.abcam.cn/products/primary-antibodies/fgfr3-antibody-epr23043-ab133644.html), FGF18 (https://www.ptgcn.com/products/FGF18-Antibody-11495-1-AP.htm), PI3K (https://www.abcam.cn/products/primary-antibodies/pi-3-kinase-p85-alpha-antibody-epr18702-ab191606.html), p-PI3K (https://www.abcam.cn/products/primary-antibodies/pi-3-kinase-p85-alpha-phospho-y607-antibody-ab182651.html), AKT (https://www.abcam.cn/products/primary-antibodies/akt1--akt2--akt3-antibody-epr16798-ab179463.html), p-AKT (https://www.abcam.cn/products/primary-antibodies/akt1--akt2--akt3-phospho-s472--s473--s474-antibody-epr18853-ab192623.html), p-AKT (https://www.ptgcn.com/products/AKT1-phospho-S473-Antibody-66444-1-Ig.htm), p-AKT (https://www.scbt.com/p/p-akt1-2-3-antibody-c-11?requestFrom=search), mTOR (https://www.abcam.cn/products/primary-antibodies/mtor-antibody-epr390n-ab134903.html), p-mTOR (https://www.abcam.cn/products/primary-antibodies/mtor-phospho-s2448-antibody-epr4262-ab109268.html), HDAC3 (https://www.abcam.cn/products/primary-antibodies/hdac3-antibody-y415-ab32369.html), LC3B (https://www.abcam.cn/products/primary-antibodies/lc3b-antibody-epr18709-autophagosome-marker-ab192890.html), SQSTM1 (https://www.abcam.cn/products/primary-antibodies/sqstm1--p62-antibody-epr4844-autophagosome-marker-ab109012.html). Alexa 488-conjugated goat anti-mouse secondary antibody (https://www.thermofisher.cn/cn/zh/antibody/product/Goat-anti-Mouse-IgG-H-L-Cross-Adsorbed-Secondary-Antibody-Polyclonal/A-11001), Alexa 555-conjugated donkey anti-rabbit secondary antibody (https://www.beyotime.com/product/A0453.htm), Goat anti-Mouse IgG (http://www.fdbio.net/productinfo.php?id=220), Goat anti-Rabbit IgG: (http://www.fdbio.net/productinfo.php?id=219), Anti Mouse/Rabbit IgG (https://www.boster.com.cn/home/product/sv-igg_sv0004.html).

Eukaryotic cell lines

Policy information about cell lines and Sex and Gender in Research

Cell line source(s)	No cell line was used in this study
Authentication	None of these cell line were authenticated by us.
Mycoplasma contamination	All cell lines tested negative for mycoplasma contamination.
Commonly misidentified lines (See ICLAC register)	No commonly misidentified cell lines were used.

Animals and other research organisms

Policy information about studies involving animals; ARRIVE guidelines recommended for reporting animal research, and Sex and Gender in Research

Laboratory animals	CicRreb1 global knockout (CircRreb1 gKO) mice were purchased from Cyagen Biosciences Inc (Suzhou, China). 18-momth-old CircRreb1 gKO mice and 18-month-old wide type C57BL/6 mice were used in this study to observe the senescent and OA phenotypes. CircRreb1 flox/flox and ACAN-CreERT2 mice were purchased from Cyagen Biosciences Inc. CircRreb1 flox/flox and ACAN-CreERT2 mice were used to bred ACAN-CreERT2 CircRreb1 fl/fl mice (CircRreb1 cKO). 8-week-old CircRreb1mice (Fig.6), 3-month-old wide type mice (Fig.3), 18-month-old wide type mice (Fig.3), and 8-week-old C57BL/6 mice (Extended Data Fig.10) were received DMM OA model The housing conditions for mice is maintained in a SPF environment. Animals were maintained under constant temperature (23-25°C), circulating air and humidity (45-65%) with a 12 h: 12 h light/dark cycle. Mice had fee access to food and water.
Wild animals	This study did not involved wild animals

Reporting on sex	To generate male CircRreb1 flox/flox; ACAN-CreERT2 mice, male CircRreb1 flox/flox mice were mated with female ACAN-CreERT2 mice to produce CircRreb1 flox/+;ACAN-CreERT2 mice, which were then mated with CircRreb1 flox/flox mice. Male mice were used for DMM model.
Field-collected samples	The study did not involve samples collected from the field.
Ethics oversight	Ethical approval was received from the Animal Care and Use Committee of Zhejiang University Health Science Center.

Note that full information on the approval of the study protocol must also be provided in the manuscript.

Flow Cytometry

Plots

Confirm that:

☒ The axis labels state the marker and fluorochrome used (e.g. CD4-FITC).

☒ The axis scales are clearly visible. Include numbers along axes only for bottom left plot of group (a 'group' is an analysis of identical markers).

☒ All plots are contour plots with outliers or pseudocolor plots.

☒ A numerical value for number of cells or percentage (with statistics) is provided.

Methodology

Sample preparation	human primary chondrocytes
Instrument	Flow cytometer (BD Biosciences)
Software	BD FACSDiva v9.0
Cell population abundance	CM-H2DCFDA was employed to quantitatively determine the percentage of cells express ROS. CM-H2DCFDA is hydrolyzed into DCFH in the cell, and DCFH is oxidized into DCF with high fluorescence intensity by the oxidant in the cell, which is used to detect the generation of ROS
Gating strategy	Cells stained with DCF were positive.

☒ Tick this box to confirm that a figure exemplifying the gating strategy is provided in the Supplementary Information.
